# Targeting LINC00152 activates cAMP/Ca^2+^/ferroptosis axis and overcomes tamoxifen resistance in ER+ breast cancer

**DOI:** 10.1038/s41419-024-06814-3

**Published:** 2024-06-15

**Authors:** Ozge Saatci, Rashedul Alam, Kim-Tuyen Huynh-Dam, Aynur Isik, Meral Uner, Nevin Belder, Pelin Gulizar Ersan, Unal Metin Tokat, Burge Ulukan, Metin Cetin, Kubra Calisir, Mustafa Emre Gedik, Hilal Bal, Ozlem Sener Sahin, Yasser Riazalhosseini, Denis Thieffry, Daniel Gautheret, Besim Ogretmen, Sercan Aksoy, Aysegul Uner, Aytekin Akyol, Ozgur Sahin

**Affiliations:** 1 Department of Biochemistry and Molecular Biology, Hollings Cancer Center, Medical University of South Carolina, Charleston, SC 29425 USA; 2 Department of Drug Discovery and Biomedical Sciences, University of South Carolina, Columbia, SC 29208 USA; 3 Department of Pathology, Faculty of Medicine, Hacettepe University, 06100 Ankara, Turkey; 4 Department of Molecular Biology and Genetics, Bilkent University, Ankara, 06800 Turkey; 5 Department of Human Genetics, McGill University, Montreal, Quebec Canada; 6 Victor Philip Dahdaleh Institute of Genomic Medicine at McGill University, Montreal, Quebec Canada; 7 Département de biologie de l’Ecole normale supérieure, PSL Université, 75005 Paris, France; 8 Bioinformatics and Computational Systems Biology of Cancer, U900 Institut Curie - INSERM - Mines ParisTech, PSL Université, 75005 Paris, France; 9 Institute for Integrative Biology of the Cell (I2BC), Université Paris-Saclay, CNRS, CEA, 91190 Gif-sur-Yvette, France; 10 Department of Medical Oncology, Hacettepe University Cancer Institute, 06100 Ankara, Turkey

**Keywords:** Breast cancer, Cancer

## Abstract

Tamoxifen has been the mainstay therapy to treat early, locally advanced, and metastatic estrogen receptor-positive (ER + ) breast cancer, constituting around 75% of all cases. However, the emergence of resistance is common, necessitating the identification of novel therapeutic targets. Here, we demonstrated that long-noncoding RNA LINC00152 confers tamoxifen resistance by blocking tamoxifen-induced ferroptosis, an iron-mediated cell death. Mechanistically, inhibiting LINC00152 reduces the mRNA stability of phosphodiesterase 4D (*PDE4D*), leading to activation of the cAMP/PKA/CREB axis and increased expression of the TRPC1 Ca^2+^ channel. This causes cytosolic Ca^2+^ overload and generation of reactive oxygen species (ROS) that is, on the one hand, accompanied by downregulation of FTH1, a member of the iron sequestration unit, thus increasing intracellular Fe^2+^ levels; and on the other hand, inhibition of the peroxidase activity upon reduced GPX4 and xCT levels, in part by cAMP/CREB. These ultimately restore tamoxifen-dependent lipid peroxidation and ferroptotic cell death which are reversed upon chelating Ca^2+^ or overexpressing GPX4 or xCT. Overexpressing PDE4D reverses LINC00152 inhibition-mediated tamoxifen sensitization by de-activating the cAMP/Ca^2+^/ferroptosis axis. Importantly, high LINC00152 expression is significantly correlated with high PDE4D/low ferroptosis and worse survival in multiple cohorts of tamoxifen- or tamoxifen-containing endocrine therapy-treated ER+ breast cancer patients. Overall, we identified LINC00152 inhibition as a novel mechanism of tamoxifen sensitization via restoring tamoxifen-dependent ferroptosis upon destabilizing PDE4D, increasing cAMP and Ca^2+^ levels, thus leading to ROS generation and lipid peroxidation. Our findings reveal LINC00152 and its effectors as actionable therapeutic targets to improve clinical outcome in refractory ER+ breast cancer.

## Introduction

Estrogen receptor-positive (ER + ) breast cancer accounts for almost 75% of all breast cancers. Endocrine therapies, modulating ER level and/or activity are the standard of care in ER+ breast cancer. The selective ER modulator (SERM) tamoxifen has been the backbone of endocrine therapy for over 40 years, especially for premenopausal women [1, 2]. Tamoxifen is recommended to treat early, locally advanced and metastatic ER+ breast cancers [3] and significantly improves overall survival [3, 4]. Despite its initial clinical success, patients inevitably develop resistance and experience poor clinical outcomes [5]. The molecular mechanisms of tamoxifen resistance are partially understood and involve ER downregulation, *ESR1* mutations, modulation of cell cycle regulators, activation of receptor tyrosine kinases and downstream effectors, and modulation of non-coding RNAs [6–10]. The clinical management of tamoxifen resistance involves the use of other endocrine therapies or CDK4/6 inhibitors [11]. However, resistance to second-line therapies is also inevitable [12]. Therefore, there is still an unmet clinical need for the identification of key processes and novel targets to overcome tamoxifen resistance and improve patient outcome.

Ferroptosis is a relatively newly discovered iron-dependent oxidative form of cell death that is characterized by lipid peroxidation, cell volume shrinkage and increased mitochondrial membrane density [13–15]. It is under tight control by the interplay between pro-oxidant and antioxidant systems [16]. Major indicators of ferroptosis are the increase in lipid peroxidation and decreased expression or activity of the glutathione peroxidase 4 (GPX4), a major component of the antioxidant system [17]. Certain anti-cancer therapies have been shown to activate ferroptosis, such as sorafenib [18], lapatinib [19], and radiation therapy [20], and vulnerability to ferroptotic cell death has been demonstrated to be a feature of certain therapy-resistant cancer cells [21]. Along these lines, ferroptosis induction is emerging as an effective strategy to eradicate therapy-resistant cancer cells. However, the potential roles of ferroptosis in tamoxifen-mediated cell death or activating ferroptosis in overcoming resistance to endocrine therapies used in ER+ breast cancer, including tamoxifen, are mostly unknown.

Long non-coding RNAs (lncRNAs) are non-coding RNAs with >200 nucleotides in length that carry out diverse functions, including transcriptional regulation, regulation of proteins or RNA molecules by direct binding and stabilization, and sponging miRNAs [22]. LncRNAs may also drive resistance to anti-cancer therapies, including endocrine therapy via protecting ER against downregulation [23], enhancing ER signaling [24] or activating downstream PI3K/AKT or mTOR signaling [25]. LINC00152 is an oncogenic lncRNA that promotes survival, proliferation, epithelial-mesenchymal transition (EMT) and invasiveness in cancer cells [26–28]. It has been associated with tumor aggressiveness [28, 29] and demonstrated to be a biomarker in various cancers, such as breast cancer, colorectal cancer, and glioma [30–32]. Despite being a driver in several key oncogenic processes, the potential functions of LINC00152 in regulating distinct forms of cell death, such as ferroptosis to drive resistance to endocrine therapy, including tamoxifen in ER+ breast cancer remain to be elucidated.

Here we showed that LINC00152 is a driver of tamoxifen resistance, and its inhibition causes tamoxifen sensitization via reducing the mRNA stability of its novel interactor *PDE4D*, a cAMP-degrading phosphodiesterase. This leads to the activation of cAMP/PKA/CREB axis, which increases the expression of the TRPC1 Ca^2+^ channel, causing cytosolic Ca^+2^ overload, generation of reactive oxygen species (ROS) and increase in lipid peroxidation, leading to restoration of tamoxifen-induced ferroptosis via deactivation of the xCT/GPX4 axis. Notably, overexpressing PDE4D rescues LINC00152-mediated tamoxifen resistance by blocking tamoxifen-induced ferroptosis.

## Results

### LINC00152 is upregulated in tamoxifen resistance, and high LINC00152 expression correlates with worse prognosis

**Fig. 1:**
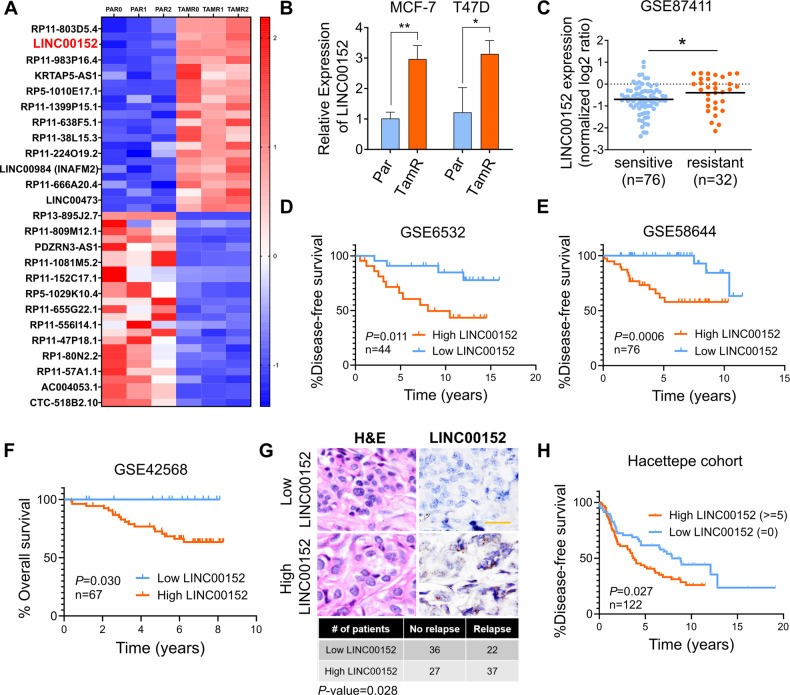
LINC00152 is upregulated in tamoxifen resistance and its higher expression correlates with poor prognosis. **A** Heatmap of the z-scores of the FPKM values of the top 25 most abundant, upregulated, and top 25, least abundant, downregulated lncRNAs in MCF-7 tamoxifen-resistant (TAMR) cells compared to parental (Par) cells in three biological replicates (0, 1, 2). The names of 25 genes are depicted on the heatmap. Red indicates upregulation and blue indicates downregulation in TamR cells. **B** qRT-PCR analysis of LINC00152 in both MCF-7 and T47D.Par and TamR cells. **C** LINC00152 levels in tamoxifen sensitive vs. resistant tumors at baseline from GSE87411. **D**, **E** The disease-free survival in tamoxifen-treated ER+ breast cancer patients with tumors expressing low vs. high LINC00152 expression from GSE6532 (**D**) and GSE58644 (**E**). **F** The overall survival in patients with tumors expressing low vs. high LINC00152 expression from GSE42568. **G** Representative in situ hybridization (ISH) staining of LINC00152 in low vs. high expressers among endocrine therapy-treated ER+ breast carcinoma specimens from the Hacettepe cohort. Scale bar = 20 µm. The numbers of Hacettepe patients having low vs high LINC00152 and with recurrence vs. no recurrence are provided at the bottom of the panel. **H** The disease-free survival in Hacettepe patients with tumors expressing low vs. high LINC00152 expression as quantified by ISH staining. Data are presented as mean values ± standard deviation (SD). *P*-values for the bar graphs and boxplots were calculated with the unpaired, two-tailed Student’s *t*-test. The significance for the Kaplan–Meier survival graphs was calculated with the Log-rank test. The chi-square test was used for (**G**). *P < 0.05, **P < 0.01.

To identify differentially expressed lncRNAs involved in tamoxifen resistance, we performed RNA-sequencing (RNA-seq) (Supplementary Fig. S1A, B) using our previously generated acquired tamoxifen-resistant (TamR) MCF-7 cells along with the parental (Par) counterparts, which were cultured alongside resistant cells without drug treatment, and therefore remained sensitive to tamoxifen [33]. Comprehensive GSEA analysis showed enrichment of tamoxifen resistance and loss of estrogen response-related genes within TamR cells (Supplementary Data 1, Supplementary Fig. S1C), validating resistance. We identified 330 differentially expressed lncRNAs (*q*-value = 0.05) between resistant and parental isogenic cells (Supplementary Data 2). LINC00152 was among the most significantly upregulated (FDR < 0.001, log2FC = 1.39), highly abundant (average FPKM ~ 25, top 5th highest among all lncRNAs), validated and oncogenic [34] lncRNAs in tamoxifen-resistant cells compared to parental counterparts (Fig. 1A, Supplementary Fig. S1B, Supplementary Data 2). We further validated its upregulation via qRT-PCR analysis in MCF-7.TamR and T47D.TamR cells, a second tamoxifen-resistant ER+ breast cancer cell line that we developed [33] compared to parental cells (Fig. 1B).

To test the clinical relevance of LINC00152 upregulation in tamoxifen resistance, we first analyzed available gene expression profiling data of tamoxifen-treated ER+ breast cancer patients. We demonstrated a significantly higher expression of LINC00152 in patients with no response to tamoxifen therapy compared to patients exhibiting tamoxifen response in a dataset with available response information, GSE87411 (Fig. 1C). A list of genes previously reported to be upregulated in tamoxifen or endocrine resistance were enriched in ER+ breast cancer patients expressing higher LINC00152 (Supplementary Fig. S1D, E). Furthermore, we demonstrated that higher LINC00152 expression is significantly associated with worse disease-free and overall survival (Fig. 1D–F). We then performed in-situ hybridization (ISH) of LINC00152 in our own cohort of endocrine therapy-treated ER+ breast cancer patients and demonstrated that higher LINC00152 levels are associated with significantly worse disease-free survival (Fig. 1G, H). The LINC00152 ISH score was also higher in tumors with high Ki67 score, which is a marker of cell proliferation [35] and high grade (Supplementary Fig. S1F, G). Moreover, we showed that LINC00152 levels are higher in tumors compared to normal tissues (Supplementary Fig. S2A, B) and that its higher expression is associated with worse overall survival (Supplementary Fig. S2C, D) also in patients with other tumors, e.g., hepatocellular carcinoma and lung adenocarcinoma. These data suggest that in addition to its roles in driving tamoxifen resistance, LINC00152 may also play key roles in tumorigenesis in general. Overall, we identified LINC00152 as a clinically relevant lncRNA upregulated in tamoxifen resistance.

### Inhibiting LINC00152 overcomes tamoxifen resistance by inducing tamoxifen-dependent ferroptosis

**Fig. 2:**
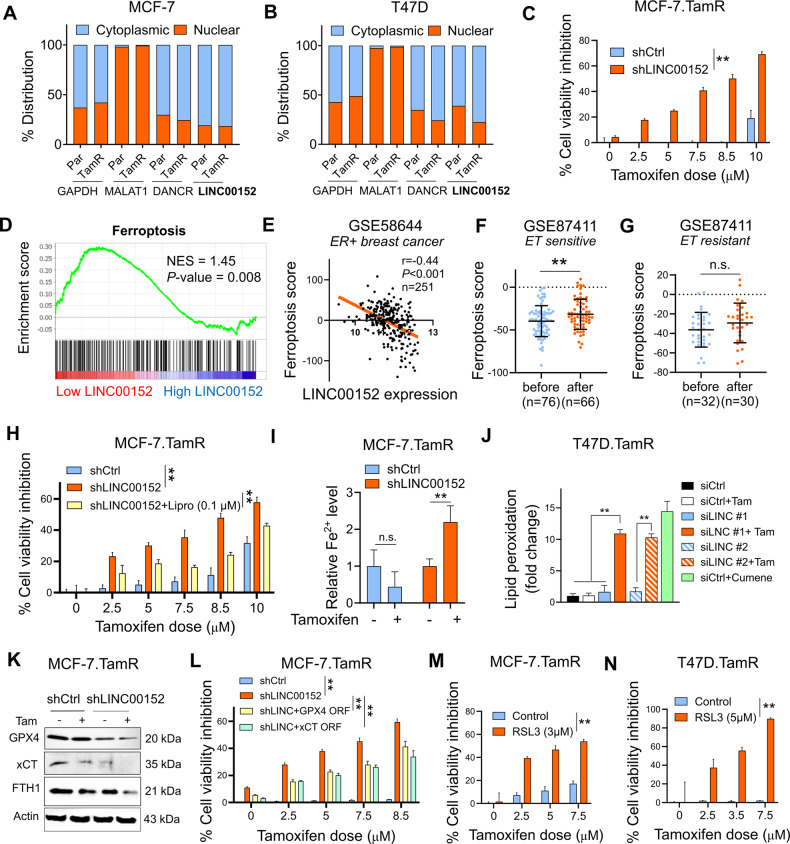
Inhibiting LINC00152 overcomes tamoxifen resistance by inducing tamoxifen-dependent ferroptosis. **A, B** The nuclear vs. cytoplasmic localization of LINC00152 in MCF-7 (**A**) and T47D (**B**) Par and TamR cells. MALAT1 and DANCR were used as controls for nuclear and cytoplasmic RNAs, respectively. **C** Percent cell viability inhibition in MCF-7.TamR cells expressing shLINC00152 upon treatment with tamoxifen. **D** GSEA analysis showing the ferroptosis-related genes enriched in low LINC00152-expressing ER+ breast cancer patients treated with endocrine therapy from GSE87411. **E** Pearson correlation analysis of LINC00152 expression with ferroptosis score in ER+ patients from GSE58644. **F**, **G** The levels of ferroptosis score before and after endocrine therapy in sensitive (**F**) and resistant (**G**) ER+ patients from GSE87411. **H** Percentage cell viability inhibition in shLINC00152-expressing MCF-7.TamR cells treated with tamoxifen with or without 0.1 µM of liproxstatin-1 (Lipro). **I** Relative intracellular Fe^2+^ levels in shCtrl or shLINC00152-expressing MCF-7.TamR cells that were treated with 7.5 µM tamoxifen for 1.5 h. **J** Lipid peroxidation assay in T47D.TamR cells transfected with siLINC00152 (siLINC) in combination with tamoxifen, showing fold change of lipid peroxidation. Cumene was used as a positive control. **K** Western blot analysis of the negative regulators of ferroptosis, xCT, GPX4, and FTH1 in MCF-7.TamR cells expressing shLINC00152 and treated with tamoxifen. Actin is used as a loading control in all Western blots unless stated otherwise. **L** Percentage cell viability inhibition in shLINC00152-expressing MCF-7.TamR cells transfected with GPX4 or xCT open reading frame (ORF). **M**, **N** Percentage cell viability inhibition in MCF-7.TamR (**M**) and T47D.TamR (**N**) cells treated with tamoxifen alone or in combination with 3 or 5 µM RSL3. Data are presented as mean values ± standard deviation (SD). *P*-values for (**D**) were calculated using the GSEA software (Broad Institute) while others were calculated with the paired (cell viability) or unpaired (others), two-tailed Student’s *t*-test. *P < 0.05, **P < 0.01, n.s. not significant.

To determine the roles of LINC00152 in tamoxifen resistance, we first determined its cellular localization. We showed that LINC00152 is primarily a cytoplasmic lncRNA in both Par and TamR MCF-7 and T47D cells (Fig. 2A, B). Here, lncRNAs, DANCR [36] and MALAT1 [37] are shown as positive controls as they are known to be cytoplasmic and nuclear, respectively. Then, we showed that stable LINC00152 knockdown (MCF-7.TamR.shLINC00152) (Supplementary Fig. S3A) led to a significant increase in cell viability inhibition in combination with tamoxifen (Fig. 2C). These results were also recapitulated by using two different siRNAs against LINC00152 (Supplementary Fig. S3B, C). On the contrary, overexpressing LINC00152 reduced the viability inhibition induced by tamoxifen (Supplementary Fig. S3D–G).

Next, to identify the molecular mechanisms of LINC00152-mediated tamoxifen resistance, we performed RNA-seq upon siRNA-mediated knockdown of LINC00152 in the MCF-7.TamR cells. We generated a LINC00152 knockdown (KD) score comprising of genes down or upregulated upon LINC00152 knockdown (0.67 > FC > 1.5, *P* < 0.1). Accordingly, patients who express high LINC00152 KD score are expected to express low levels of LINC00152. Tamoxifen sensitivity-related genes were enriched among patients who express high LINC00152 KD score, i.e., showing low LINC00152 expression, supporting the role of LINC00152 in tamoxifen resistance (Supplementary Fig. S4A). Then, we performed pathway enrichment analysis and observed a significant enrichment of genes involved in ferroptosis among LINC00152-modulated mRNAs (Supplementary Fig. S4B). Gene set enrichment analysis (GSEA) among endocrine therapy-treated ER+ breast cancer patients separated based on LINC00152 expression revealed significant enrichment of a validated ferroptosis gene signature, generated using FerrDb V2 database [38] in patients with low LINC00152 expression (Fig. 2D). Furthermore, several ferroptosis-related pathways e.g., glutathione metabolism, sphingolipid pathway and selenoamino acid metabolism were enriched (Supplementary Fig. S4C). We observed a significant negative correlation between LINC00152 expression and ferroptosis signature score among ER+ breast cancer patients in an independent dataset (Fig. 2E). Furthermore, there was a significant increase in the ferroptosis score in sensitive tumors, but not in resistant ones, after treatment with endocrine therapy in the GSE87411 dataset in which samples were collected before and after endocrine therapy (Fig. 2F, G), suggesting that ferroptosis induction is associated with clinical endocrine therapy sensitivity.

To experimentally test the role of ferroptosis induction in LINC00152 inhibition-mediated tamoxifen sensitization, we inhibited ferroptosis using the ferroptosis inhibitor liproxstatin-1 [39] and showed partial rescue of cell viability (Fig. 2H). Furthermore, knockdown of LINC00152 in combination with tamoxifen significantly increased intracellular Fe^2+^ levels (Fig. 2I) that were followed by lipid peroxidation (Fig. 2J), and deactivation of xCT, GPX4, and FTH1 (Fig. 2K), major antioxidant proteins [17, 40] or iron chelators [41, 42]. Importantly, overexpressing xCT or GPX4 reduced LINC00152 inhibition-mediated tamoxifen sensitization (Fig. 2L, Supplementary Fig. S5A, B). Supporting these results, knocking down xCT or GPX4 resulted in the reversal of LINC00152-mediated tamoxifen resistance that was accompanied by reduction of LINC00152-induced xCT/GPX4 levels (Supplementary Fig. S5C–F). Likewise, inhibiting GPX4 pharmacologically using RSL3 phenocopied the effect of LINC00152 knockdown on tamoxifen sensitization (Fig. 2M, N). To test if ferroptosis is a mechanism of tamoxifen-induced cell death in parental cells, we inhibited ferroptosis using liproxstatin-1 which partially rescued cell viability in parental cells under tamoxifen treatment in a dose-dependent manner (Supplementary Fig. S6A, B). Tamoxifen-induced cell viability inhibition was also reversed by vitamin E, a lipid soluble antioxidant with anti-ferroptosis activity [43] (Supplementary Fig. S6C). Supporting these results, tamoxifen reduced the expression of xCT and FTH1, and increased PTGS2, a marker of ferroptosis in parental cells (Supplementary Fig. S6D). Of note, while inhibitors of necrosis (Necrox-5) or necroptosis (GSK-872) did not alter tamoxifen sensitivity, inhibiting apoptosis, using Q-Vd-OPh partially rescued cell viability under treatment with tamoxifen (Supplementary Fig. S7A-C). Markers of apoptosis such as p21, c. PARP and c. caspase 7 were also induced by tamoxifen in parental cells (Supplementary Fig. S7D). This is in line with previous studies showing apoptotic cell death as one of the mechanisms of action of tamoxifen [44]. Overall, our data suggest that LINC00152 confers tamoxifen resistance in part via counteracting tamoxifen-induced ferroptosis, thus rendering TamR cells dependent on LINC00152, whose inhibition restores tamoxifen-induced ferroptosis and inhibition of cell viability.

### LINC00152 inhibition increases TRPC1 expression and cytosolic Ca^2+^ downstream of cAMP signaling

**Fig. 3:**
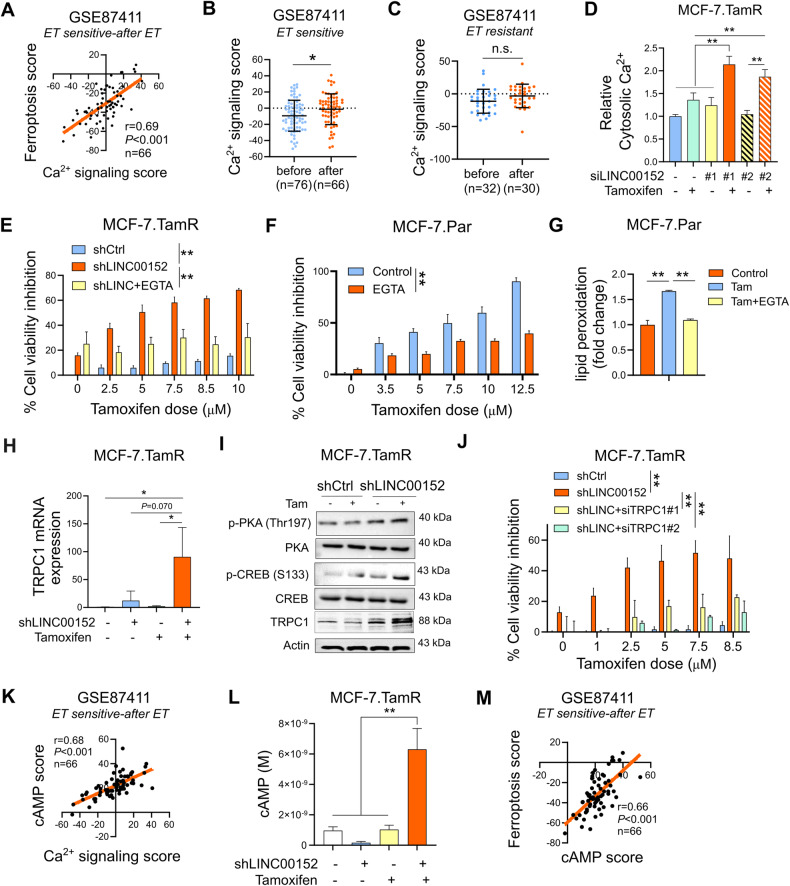
LINC00152 inhibition increases TRPC1 expression and cytosolic Ca^2+^ downstream of cAMP signaling. **A** The Pearson correlation analysis of Ca^2+^ signaling with ferroptosis score in endocrine therapy sensitive ER+ patients after therapy from GE87411. **B, C** The levels of Ca^2+^ signaling score before and after endocrine therapy in sensitive (**B**) and resistant (**C**) ER+ patients from GSE87411. **D** The levels of cytosolic Ca^2+^ in MCF-7.TamR cells that were transfected with two different siRNAs against LINC00152 and treated with tamoxifen. **E** Percentage cell viability in shLINC00152-expressing MCF-7.TamR cells treated with tamoxifen with or without EGTA. **F** Percentage cell viability inhibition in MCF-7.Par cells treated with tamoxifen with or without EGTA. **G** Relative lipid peroxidation in cells from (**F**). **H** qRT-PCR analysis of *TRPC1* mRNA in shCtrl or shLINC00152-expressing MCF-7.TamR cells treated with tamoxifen. **I** Western Blot analysis of PDE4D, p-CREB (S133), p-PKA (Thr197) and TRPC1 in shCtrl or shLINC00152-expressing MCF-7.TamR cells treated with tamoxifen. **J** Percentage cell viability in shLINC00152-expressing MCF-7.TamR cells transfected with 2 different siRNAs against TRPC1. **K** The Pearson correlation analysis of Ca^2+^ signaling score with cAMP score in endocrine therapy sensitive ER+ patients after therapy from GSE124647. **L** cAMP levels in MCF-7.TamR shCtrl or shLINC00152 cells treated with tamoxifen. **M** The Pearson correlation analysis of cAMP score and ferroptosis score in endocrine therapy sensitive ER+ patients after therapy from GSE87411. Data are presented as mean values ±standard deviation (SD). *P*-values were calculated with the paired (cell viability) or unpaired (others), two-tailed Student’s *t*-test. *P < 0.05, **P < 0.01, n.s. not significant.

Increase in cytosolic Ca^2+^ has been shown to be a major regulator of ferroptosis [45]. In line with this, we found a significant positive correlation between the scores of validated Ca^2+^ signaling [46] and ferroptosis [38] signatures in post endocrine therapy-treated sensitive ER+ breast cancer patients (Fig. 3A). Ca^2+^ signaling score was also significantly upregulated after endocrine therapy in sensitive but not in resistant patients (Fig. 3B, C), like the ferroptosis score in the same patient population (Fig. 2F, G). Supporting these results, we observed a significant increase in Ca^2+^ levels in siLINC00152-transfected MCF-7.TamR cells treated with tamoxifen (Fig. 3D). Importantly, chelating Ca^2+^ using EGTA reversed LINC00152 inhibition-induced tamoxifen sensitization (Fig. 3E). Notably, EGTA treatment also reduced tamoxifen-induced viability inhibition via inhibiting tamoxifen-induced ferroptosis (Fig. 3F, G), suggesting that Ca^2+^ induction is one of the key mechanisms of tamoxifen-induced ferroptosis and sensitivity, which is restored in TamR cells upon LINC00152 inhibition.

To identify the regulator of Ca^2+^ signaling under the control of LINC00152, we analyzed the RNA-seq data of siLINC00152-transfected cells and found that TRPC1, a Ca^2+^ channel located on cell or endoplasmic reticulum (EnR) membrane and regulating the Ca^2+^ levels within the cytosol [47], was upregulated upon LINC00152 knockdown (Supplementary Fig. S8A). This was validated at mRNA and protein levels by qRT-PCR and Western blot, respectively, and its level was further increased in combination with tamoxifen in TamR cells (Fig. 3H, I). Importantly, knocking down TRPC1 using two different siRNAs rescued cell viability under tamoxifen treatment (Fig. 3J, Supplementary Fig. S8B), while overexpressing TRPC1 in TamR cells increased tamoxifen sensitivity (Supplementary Fig. S8C, D), showing the functional role of TRPC1 in LINC00152 inhibition-mediated tamoxifen sensitization.

Increase in cAMP is known to stimulate Ca^2+^ flux by activating the calcium channels [48]. We identified a significant positive correlation between calcium signaling score and cAMP signaling score [49] after treatment with endocrine therapy (Fig. 3K) in the same endocrine-sensitive patients from GSE87411 showing positive correlation between Ca^2+^ signaling and ferroptosis scores (Fig. 3A). Supporting these results, we detected a significant increase in cAMP upon knocking down LINC00152 in combination with tamoxifen (Fig. 3L) that was followed by phosphorylation of the downstream effectors, PKA and CREB (Fig. 3I). Notably, inhibiting CREB rescued the expression of xCT in shLINC00152-expressing MCF-7.TamR cells (Supplementary Fig. S8E) and tamoxifen-treated parental cells (Supplementary Fig. S8F), suggesting that cAMP/CREB is not only important for Ca^2+^/ROS induction but also for suppressing xCT/GPX4 to promote LINC00152 inhibition-induced and tamoxifen-dependent ferroptosis. Furthermore, the levels of cAMP score were significantly positively correlated with the ferroptosis score in post-endocrine therapy-treated sensitive ER+ breast cancer patients (Fig. 3M), similar to calcium signaling score (Fig. 3A). Altogether, these data demonstrate that LINC00152 inhibition in combination with tamoxifen activates cAMP signaling and increases TRPC1 levels, thus triggering cytoplasmic Ca^2+^ overload that likely initiates ferroptosis in TamR cells.

### LINC00152 inhibits tamoxifen-dependent ferroptosis by regulating PDE4D and downstream cAMP/Ca^2+^ signaling in tamoxifen-resistant cells

**Fig. 4:**
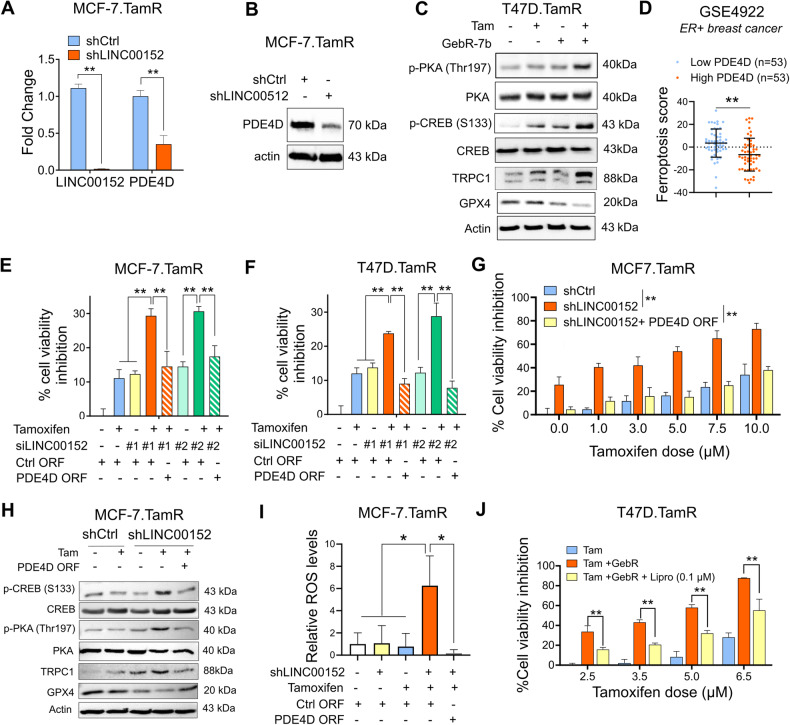
LINC00152 inhibits tamoxifen-dependent ferroptosis by regulating PDE4D and downstream cAMP/Ca^2+^ signaling in tamoxifen-resistant cells. **A** qRT-PCR analysis of LINC00152 and PDE4D in the shLINC00152-expressing MCF-7.TamR cells. **B** Western blot analysis of PDE4D in the shLINC00152-expressing MCF-7.TamR cells. **C** Western blot analysis of p-CREB (S133), p-PKA (Thr197), TRPC1 and ferroptosis inhibitor GPX4 in T47D.TamR cells that were treated with tamoxifen in combination with GebR-7b. **D** The levels of ferroptosis score in low vs. high PDE4D-expressing ER+ patients from GSE4922. **E**, **F** Percentage cell viability inhibition in MCF-7 and T47D.TamR cells co-transfected with siLINC00152 and PDE4D ORF and treated with tamoxifen. **G** Percentage of viability inhibition in shLINC00152-expressing MCF-7.TamR cells stably overexpressing PDE4D and that were treated with tamoxifen. **H** Western blot analysis of p-CREB (S133), p-PKA (Thr197), TRPC1 and GPX4 in shLINC00152-expressing MCF-7.TamR cells stably overexpressing PDE4D and that were treated with tamoxifen. **I** Relative ROS levels in MCF-7.TamR cells expressing shLINC00152 and treated with tamoxifen. **J** Percentage viability inhibition in T47D.TamR cells treated with tamoxifen and GebR-7b with or without 0.1 µM of liproxstatin-1 (Lipro). Data are presented as mean values ± standard deviation (SD). *P*-values were calculated with the paired (cell viability) or unpaired (others), two-tailed Student’s *t*-test. *P < 0.05, **P < 0.01.

Phosphodiesterase 4D (PDE4D) is one of the cAMP-specific phosphodiesterases that degrades cAMP [50]. We found that *PDE4D* mRNA is downregulated in the RNA-seq data of siLINC00152-transfected MCF-7.TamR cells (Supplementary Fig. S9A). The reduction was further validated at both mRNA and protein levels by qRT-PCR and Western blotting, respectively, upon siRNA or shRNA-mediated LINC00152 knockdown in TamR cells (Fig. 4A, B, Supplementary Fig. S9B, C). Conversely, overexpressing LINC00152 in parental MCF-7 and T47D cells significantly increased the mRNA and protein levels of PDE4D (Supplementary Fig. S9D, E). Notably, combining tamoxifen with the PDE4D inhibitor GebR-7b [51] increased TRPC1 levels along with activation of PKA/CREB and reduction of GPX4 (Fig. 4C), phenocopying the effects of LINC00152 knockdown (Fig. 3I). Next, we demonstrated a significantly lower ferroptosis score in patients expressing high levels of PDE4D (Fig. 4D), showing the clinical relevance.

We showed that PDE4D overexpression completely rescued cell viability in the presence of siRNA or shRNA-mediated LINC00152 knockdown in combination with tamoxifen (Fig. 4E–G) via reducing TRPC1 expression along with de-activation of PKA and CREB and increased expression of GPX4 (Fig. 4H). These data demonstrate the key contribution of PDE4D in LINC00152 mediated tamoxifen resistance. Importantly, we detected a strong induction of ROS, a crucial initiator of ferroptosis [42], upon LINC00152 knockdown in combination with tamoxifen, which was completed reversed upon PDE4D overexpression (Fig. 4I). Along these lines, inhibiting ferroptosis using liproxstatin-1 partially rescued cell viability under treatment with tamoxifen and GebR-7b (Fig. 4J). Overall, these data suggest that LINC00152 blocks tamoxifen-induced ferroptosis and mediates tamoxifen resistance via regulating PDE4D/cAMP/Ca^2+^ signaling axis.

### LINC00152 stabilizes *PDE4D* mRNA by interacting with its 3’UTR

**Fig. 5:**
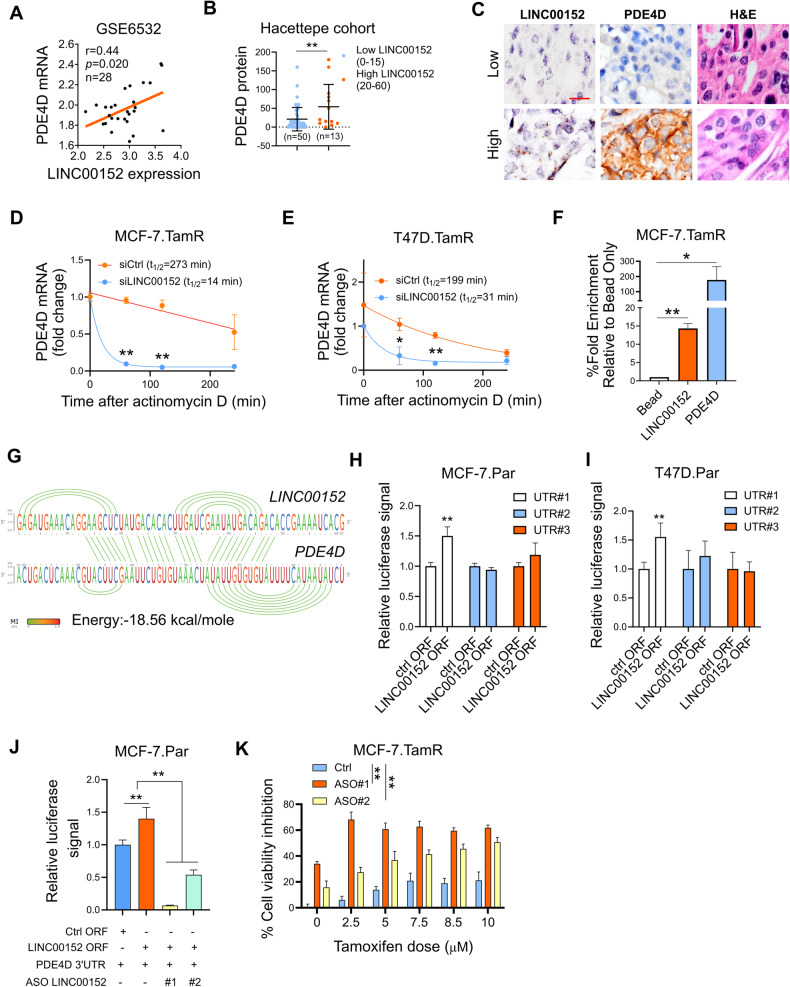
LINC00152 stabilizes *PDE4D* mRNA by interacting with its 3’UTR. **A** The Pearson correlation analysis of LINC00152 and *PDE4D* mRNA in patients from GSE6532. **B** PDE4D protein expression determined by IHC in low vs. high LINC00152-expressing patients from the Hacettepe cohort. **C** Representative images of LINC00152 ISH, PDE4D IHC, and H&E staining in patients from the Hacettepe cohort. Scale bar = 20 µm. **D**, **E** qRT-PCR analysis of PDE4D in MCF-7.TamR (**D**) and T47D.TamR (**E**) cells transfected with siLINC00152 for 48 h, followed by treatment with 5 µg/ml of actinomycin (**D**). **F** Percentage fold enrichment of PDE4D in LINC00152 pull down in MCF-7.TamR cells. **G** Interaction between LINC00152 and *PDE4D* 3’UTR predicted by the IntaRNA tool. The visualization of the interaction was obtained using RILogo. **H**, **I** Luciferase assay in parental MCF7 (**H**) and T47D (**I**) cells co-transfected with LINC00152 overexpression vector and *PDE4D* 3’UTR, demonstrating binding to *PDE4D* 3’UTR. **J** Luciferase assay in MCF-7.Par cells co-transfected with LINC00152 overexpression vector and *PDE4D* 3’UTR with or without ASOs targeting the interaction site between LINC00152 and PDE4D 3’UTR. **K** Percent cell viability inhibition in ASO-transfected MCF-7.TamR cells under tamoxifen treatment. Data are presented as mean values ± standard deviation (SD). *P*-values were calculated with the paired (cell viability) or unpaired (others), two-tailed Student’s *t*-test. *P < 0.05, **P < 0.01.

lncRNAs can modulate gene expression in multiple ways, one of which is to regulate the stability of the target mRNA [52]. We observed a positive correlation between LINC00152 and *PDE4D* mRNA in ER+ breast cancer patients progressed upon tamoxifen therapy in GSE6532 dataset, suggesting mRNA level regulation of PDE4D by LINC00152 (Fig. 5A). PDE4D also showed a positive correlation with LINC00152 at the protein level in our own cohort of ER+ breast cancer patients (Fig. 5B, C). mRNA stability assay demonstrated that LINC00152 knockdown reduces the half-life of *PDE4D* mRNA in both MCF-7 and T47D.TamR cells (Fig. 5D, E). Furthermore, RNA pull-down demonstrated the direct binding between LINC00152 and *PDE4D* mRNA in TamR cells (Fig. 5F). The 3’UTR region of mRNAs is usually responsible for regulating the stability of mRNAs [53]. Therefore, we reasoned that LINC00152 is potentially interacting with the 3’UTR region of the *PDE4D* mRNA to modulate its stability. To test this hypothesis, we first examined the predicted binding sites between *PDE4D* 3’UTR and LINC00152 using the IntaRNA tool 2.0 [54] and identified three potential interaction sites on the *PDE4D* 3’UTR (Fig. 5G, Supplementary Fig. S10). We then cloned the 3’UTR of *PDE4D* in 3 different pieces, each containing one predicted binding site for LINC00152 (UTR#1, 2, 3) downstream of the luciferase gene. Both MCF-7 and T47D cell lines were co-transfected with *PDE4D* 3’UTR vectors together with LINC00152 overexpression vector. This resulted in an increase in luciferase signal from UTR#1 (Fig. 5H, I), suggesting that LINC00152 potentially binds to 3’UTR region#1 (binding energy: −18.56 kcal/mol, Fig. 5G) and increases *PDE4D* mRNA stability. Supporting these results, two different ASO sequences that block the interaction site between LINC00152 and PDE4D 3’UTR reduced the binding between LINC00152 and PDE4D 3’UTR (Fig. 5J) and mediated tamoxifen sensitization in a dose-dependent manner (Fig. 5K). Overall, these data demonstrate that LINC00152 interacts with *PDE4D* 3’ UTR in TamR cells, increasing its mRNA stability and thereby mediating tamoxifen resistance.

### LINC00152 inhibition overcomes tamoxifen resistance in vivo

**Fig. 6:**
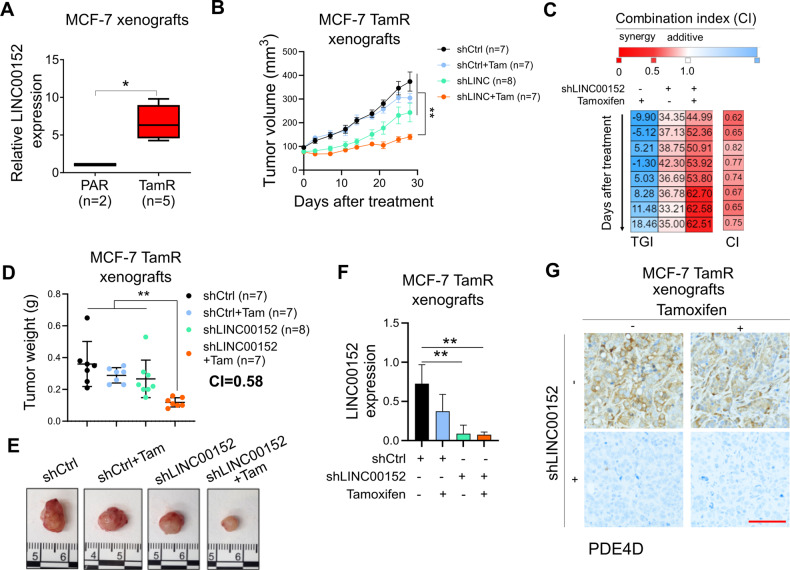
LINC00152 inhibition overcomes tamoxifen resistance in vivo. **A** *PDE4D* mRNA expression in MCF-7.Par (PAR) vs. TamR xenograft tumors. **B** Tumor volumes of MCF-7.TamR xenografts expressing shLINC00152 and treated with tamoxifen (3 mg/kg, daily). **C** Combination indices showing synergy between LINC00152 knockdown and tamoxifen in terms of tumor growth inhibition. **D** Tumor weights from mice in B. Combination index showing synergy between LINC00152 knockdown and tamoxifen in terms of tumor weight reduction is provided. **E** Tumor images from mice in (**B**). **F** qRT-PCR analysis of LINC00152 in tumors from (**B**). **G** IHC staining of PDE4D in tumors from (**B**). Scale bar = 100 µm. Data for the bar graphs and box plots are represented as mean values ± SD, while data for the tumor volume graph are represented as mean values ± standard error of the mean (SEM). *P*-values for the bar graphs and box plots were calculated with the unpaired, two-tailed Student’s *t*-test. The significance of the tumor volume graph was calculated with two-way ANOVA. *P < 0.05, **P < 0.01.

To test the effects of LINC00152 knockdown on in vivo tamoxifen sensitization, we first analyzed the expression of LINC00152 in MCF-7.TamR vs. parental tumors grown in mice and observed a significant upregulation of LINC00152 also within in vivo-grown MCF-7.TamR xenografts (Fig. 6A) like cultured cells (Fig. 1B). We then generated xenografts of MCF-7.TamR.shCtrl and shLINC00152 cells and treated the mice with vehicle (corn oil) or tamoxifen (3 mg/kg, daily) for a month. As shown in Fig. 6B–E, there was a significant synergistic reduction of tumor growth and tumor weight upon LINC00152 knockdown in combination with tamoxifen. qRT-PCR analysis of LINC00152 and immunohistochemistry (IHC) staining of PDE4D in tumors collected at the end of the experiment demonstrated that LINC00152 knockdown was sustained in vivo (Fig. 6F) and that PDE4D expression was strongly downregulated (Fig. 6G), validating our in vitro findings. Overall, we showed that LINC00152 inhibition overcomes tamoxifen resistance in vivo, at least in part, via downregulating PDE4D expression.

## Discussion

**Fig. 7:**
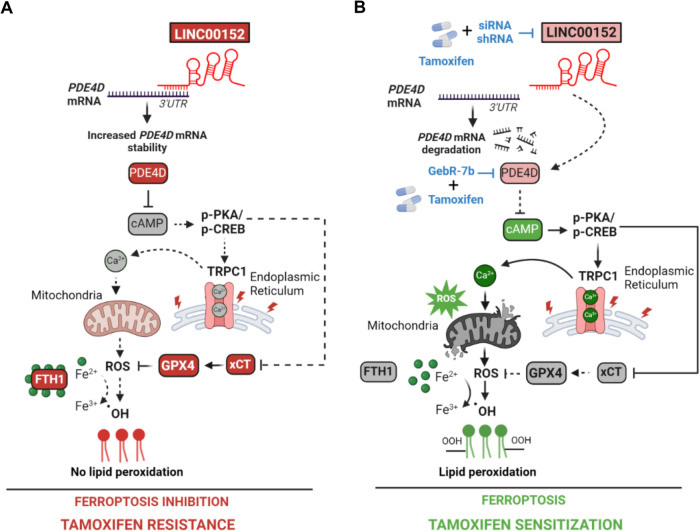
Schematic summary of LINC00152/PDE4D-driven tamoxifen resistance. **A** LINC00152 is overexpressed in tamoxifen resistance, binds to *PDE4D* 3’UTR, and increases its mRNA stability. This results in reduced cAMP levels and de-activation of PKA/CREB, leading to decreased expression of TRPC1, reduction of cytosolic Ca^2+^, and blockage of reactive oxygen species (ROS) generation. Furthermore, the negative regulators of ferroptosis, xCT, GPX4, and FTH1, are upregulated, leading to increased intracellular Fe^2+^ and inhibition of lipid peroxidation that ultimately blocks tamoxifen-dependent ferroptosis and mediates tamoxifen resistance. **B** Targeting LINC00152 in combination with tamoxifen leads to reduced mRNA stability of PDE4D due to loss of binding to its 3’UTR, thereby activating cAMP/PKA/CREB, similar to targeting PDE4D with GebR-7b in combination with tamoxifen. This further increases TRPC1 levels, elevates cytosolic Ca^2+^, generates reactive oxygen species (ROS), and increases Fe^2+^ upon downregulation of xCT, FTH1, and GPX4, leading to lipid peroxidation and tamoxifen-dependent ferroptosis. Dashed lines indicate that the connection is not active.

Tamoxifen is the first ER modulator approved by the FDA in the 1970s, and it has been the backbone of adjuvant hormone therapy [2]. However, resistance develops in ~20–30% of high-risk, advanced ER-positive breast cancer patients, decreasing survival [55]. Therefore, there is a dire need to identify novel therapeutic strategies to improve the clinical outcome for patients with resistance to tamoxifen. In this study, we demonstrated that ferroptosis is a mechanism of tamoxifen-induced cell death which is abrogated in resistance. We found that LINC00152 is overexpressed in tamoxifen resistant cells and confers tamoxifen resistance by inhibiting tamoxifen-induced ferroptosis while its inhibition confers sensitivity to tamoxifen-induced ferroptosis. We showed that LINC00152 enhances the mRNA stability of its novel target *PDE4D*, thus de-activating cAMP signaling and Ca^2+^ release and inducing xCT/GPX4, leading to inhibition of tamoxifen-dependent ferroptosis (Fig. 7A). On the other hand, LINC00152 inhibition destabilizes PDE4D and activates cAMP/PKA/CREB, like targeting PDE4D with GebR-7b. This increases TRPC1, cytosolic Ca^2+^, ROS and Fe^2+^ levels, accompanied by reduced levels of the antioxidant proteins FTH1, GPX4, and xCT, ultimately leading to lipid peroxidation, ferroptotic cell death, and tamoxifen sensitization (Fig. 7B). Notably, we showed that high LINC00152 expression is significantly correlated with high PDE4D/low ferroptosis and worse survival in multiple cohorts of tamoxifen- or tamoxifen-containing endocrine therapy-treated ER+ breast cancer patients, thereby demonstrating the clinical relevance of the axis we identified.

Activation of ferroptosis is initiated by an imbalance between prooxidant and antioxidant systems [16] and is emerging as an attractive strategy to overcome resistance to certain drugs, such as cisplatin [56, 57]. The ROS-mediated lipid peroxidation is one of the key steps driving ferroptosis upon oxidative degradation of lipids and the subsequent membrane damage [42]. On the other hand, xCT and GPX4 inhibit ferroptosis by promoting the synthesis of reduced glutathione (GSH), the primary antioxidant molecule [40], and reducing phospholipid hydroperoxide production [17], respectively. The current ferroptosis-inducing strategies used to eliminate therapy-resistant cancer cells usually target GPX4 by downregulating its expression [58] or by inhibiting its activity via the reduction of its cofactor, GSH [59], thus disturbing the balance between prooxidant and antioxidant systems. Notably, we showed that our novel combination of tamoxifen and LINC00152 knockdown induces tamoxifen-dependent ferroptosis by not only deactivating the antioxidant defense via reducing xCT and GPX4, but also by increasing Ca^2+^ overload through modulating the PDE4D/cAMP/PKA/CREB axis, thus causing ROS generation, accompanied by loss of the iron chelator, FTH1 that ultimately increases Fe^2+^ levels, and results in lipid peroxidation. Furthermore, we recently showed that endocrine therapy and CDK4/6 inhibitors commonly increase ROS levels in cancer cells and lead to cAMP-induced DNA damage, leading to PARP trapping and transcriptional blockage [60]. Future studies may investigate whether sensitivity to ferroptosis-mediated cell death is also altered in resistance to other endocrine therapies or CDK4/6 inhibitors, and whether LINC00152 targeting could also be used as an effective strategy to overcome resistance to these agents or in other resistant models vulnerable to ferroptosis induction.

Elevated levels of second messengers, such as cAMP and Ca^2+^, are known to be involved in stress response, controlling cell survival and/or death [61, 62]. The EnR is the center for managing cellular stress [63] and is the main storage site for Ca^+2^ [64]. A few anti-cancer agents are known to mediate cell death via activating EnR stress, such as bortezomib [65] and cisplatin [66]. However, the mechanisms of stress response induction in endocrine resistance are mostly undiscovered. We previously reported a novel mechanism of tamoxifen sensitization via inducing EnR stress, leading to activation of p38/JNK signaling and apoptosis in tamoxifen-resistant ER+ breast cancer cells [33]. Here, we characterized yet another novel type of stress response leading to tamoxifen sensitization, involving Ca^2+^ overload upon LINC00152/PDE4D inhibition. Recent evidence suggests that manipulating Ca^2+^ homeostasis stands as an attractive strategy to restore ferroptosis sensitivity. For instance, a novel tetraspanin, MS4A15, located on EnR membrane, blocks ferroptosis via depleting luminal Ca^2+^ stores and reprogramming lipids [67]. We demonstrated that tamoxifen-induced ferroptosis is dependent on elevated intracellular Ca^2+^ levels, and that chelating Ca^2+^ reverses tamoxifen sensitivity. In TamR cells, we identified TRPC1, a Ca^2+^ channel that can be located on a cell or EnR membrane [47], as a potentially novel regulator of ferroptosis. We showed that TRPC1 is upregulated upon activation of cAMP/PKA/CREB under LINC00152 knockdown, increases cytosolic Ca^2+^ and ROS levels. While inhibition of TRPC1 using siRNAs reverses LINC00152 inhibition-mediated tamoxifen sensitization, its overexpression confers tamoxifen sensitivity. Importantly, EnR is known to be in crosstalk with mitochondria [68], and an excessive flux of Ca^2+^ from EnR to mitochondria can lead to increased respiratory chain activity and production of excessive ROS [69], supporting our findings. Of note, since excessive Ca^2+^ may also trigger apoptotic cell death [70], elevated Ca^2+^ levels may also partially be responsible for tamoxifen-induced apoptosis. Although TRPC1 has not been reported as a CREB-induced gene, knockdown of CREB was shown to downregulate one of the G-protein coupled dopamine receptors responsible for Ca^2+^ influx, thereby reducing calcium signaling [69]. This raises the possibility that TRPC1 could also be transcribed in a CREB-dependent manner upon downregulation of LINC00152/PDE4D. Furthermore, the complete rescue of cell viability upon overexpressing PDE4D, in the presence of LINC00152 knockdown and tamoxifen, by reduction of TRPC1 and blockage of ROS, suggest that PDE4D is a novel and crucial modulator of tamoxifen-dependent ferroptotic cell death.

We demonstrated the clinical relevance of the LINC00152/PDE4D/cAMP/Ca^2+^/ferroptosis axis and showed that higher LINC00152 levels are associated with worse survival in multiple cohorts of endocrine therapy-treated ER+ breast cancer patients as well as other cancers. These results suggest that LINC00152 could be a potential biomarker for prognosis. However, future studies are warranted to test its sensitivity and specificity as well as its clinical association with the processes in our axis with larger cohorts of patients. In addition to being a potential biomarker, LINC00152 could also be targeted in clinics. LncRNAs are highly attractive targets for cancer therapy given their tissue-specific expression, thus enabling specific targeting of tumors [71]. Importantly, in the last decade, RNA-based therapies, such as ASOs or siRNAs, have undergone substantial improvements and are now being tested in clinical trials for various diseases, including cancer [72]. Overall, our data not only identify a novel mechanism of tamoxifen sensitization involving the regulation of ferroptosis by LINC00152 but also demonstrate the potential of LINC00152 as a clinically relevant resistance mediator, thus encouraging the clinical testing of LINC00152 targeting to overcome tamoxifen resistance.

## Materials and methods

### Cell lines, drugs, and culture conditions

Human breast cancer cell lines, MCF-7 and T47D, were purchased from ATCC and were cultured in phenol red–free DMEM (Gibco) with 10% FBS, 0.1% insulin, 50 U/mL penicillin/streptomycin, 1% nonessential amino acids (Gibco). Tamoxifen-resistant (TamR) MCF-7 and T47D cells were generated previously [33]. The parental counterparts (Par) were cultured alongside resistant cells without drug treatment, and therefore remained sensitive to tamoxifen. Tamoxifen and GebR-7b were purchased from Sigma. Liproxstatin-1, CREB inhibitor 666-15, Vitamin E, GSK-872, Q-Vd-OPh, and RSL3 were purchased from MedChem Express, and Necrox-5 was purchased from Cayman Chemicals. Cells were routinely tested for mycoplasma contamination using MycoAlert detection kit (Lonza) and were authenticated by STR sequencing.

### ER+ human tumor samples

To analyze the effects of LINC00152 expression on the survival of endocrine therapy-treated female ER+ breast cancer patients and its correlation with PDE4D protein, we performed in situ hybridization (ISH) of LINC00152 and immunohistochemistry (IHC) staining of PDE4D in primary tumor samples from 124 ER+ breast cancer patients that were diagnosed between 2001 and 2018 at Hacettepe University School of Medicine, Ankara, Turkey and treated with endocrine therapy with or without radiotherapy and chemotherapy. The study was approved by the Non-Interventional Clinical Research Ethics Committee of Hacettepe University (approval no: 2020/02-40).

### Whole-transcriptome sequencing (RNA-seq) and data analysis

RNA-seq of MCF-7.Par vs. TamR cells were performed in triplicates using the Illumina HiSeq 2000 platform at McGill University and Genome Quebec Innovation Centre as described previously [33]. The 330 differentially expressed lncRNAs between parental vs. TamR cells (*q*-value = 0.05) were retrieved (Supplementary Data 2). RNA-seq of MCF-7.TamR cells transfected with siControl, siLINC00152#1, or siLINC00152#2 were performed for each condition in duplicates at the University of South Carolina Functional Genomics Core. The genes differentially expressed upon LINC00152 knockdown (siControl vs. siLINC00152#1 and/or siControl vs. siLINC00152#2) with the cut-off of 0.67 > FC > 1.5 and *P* < 0.1 were determined. The details are provided in Supplementary Methods.

### Lipid peroxidation assay

For measuring the levels of lipid peroxidation as an indicator of ferroptosis, Image-iT™ Lipid Peroxidation Kit (ThermoFisher) was utilized following manufacturer’s protocol. Cells that were stained with the BODIPY™ 581/591 C11 dye provided in the kit for 30 min were analyzed by flow cytometry. The shift from red to green signal that occurs upon oxidation by lipid hydroperoxides were quantified by taking the ratio of the mean fluorescence emission at ∼590 nm to ∼510 nm and plotted as the relative ferroptosis levels.

### Cytosolic Ca^2+^ measurement

To detect cytosolic Ca^2+^ levels, the MCF-7.TamR cells were co-transfected with two different siLINC00152 and the GFP-based Ca^2+^ indicator, GCaMP3 (Addgene #64853) in 96-well format. One day later, cells were treated with 10 µM tamoxifen for 10 min, and the fluorescence signal was measured with SpectraMax microplate reader.

### RNA pulldown

T47D.TamR cells were lysed in freshly prepared lysis buffer with the addition of RNase inhibitor and Protease inhibitors (20 mM Tris-HCl, pH 7.4, 200 mM KCl, 150 mM NaCl, 1% Triton-X-100, 0.1% SDS, 1 mM DTT, protease inhibitor cocktail and 200 U/mL of Riboblock). 1 mg of total lysate was mixed with 100 pmol of biotinylated probe against LINC00152 (Supplementary Table S1) and incubated for 1 h at room temperature. Then, the lysates were incubated with blocked streptavidin beads and incubated overnight at 4 °C. The next day, beads were incubated with Proteinase K buffer (10 mM Tris-HCl pH 7.0, 100 mM NaCl, 1 mM EDTA, 0.5% SDS) and 5 μL of proteinase-K (20 mg/mL) at 56 °C for 1 h and boiled at 95 °C for 5 min. RNA was extracted via an RNA purification kit (Zymo, R1013), converted to cDNA, and analyzed by RT-qPCR using the primers listed in Supplementary Table S2. %Input method was used to calculate enrichment relative to input samples and normalized to only bead control.

### Intracellular cAMP measurements

To measure intracellular cAMP levels, MCF-7.TamR.shLINC cells that were treated with tamoxifen for 30 min were washed once with PBS and detached using Tryple. Three thousand cells/well were collected in Eppendorf tubes and lysed with ice-cold 2.5 mol/L perchloric acid, followed by neutralization with 2 M KOH. Cells were incubated on ice for 30 min and then centrifuged at 10,000 rpm for 10 min to collect the clear lysate. Twenty microliters of the clear lysate were transferred per well of a white plate, and cAMP measurement was done using the LANCE Ultra cAMP detection kit (PerkinElmer) according to the manufacturer’s protocol.

### Intracellular ROS measurement

The intracellular ROS was measured using the DCFDA / H2DCFDA - Cellular ROS Assay Kit from Abcam according to the manufacturer’s protocol. Briefly, 8000 MCF-7.TamR.shCtrl and shLINC00152 cells were seeded on clear bottom, black wall plates and, the next day, transfected with PDE4D ORF. One day later, cells were treated with tamoxifen for 9 h, followed by staining with 20 µM DCFDA for 1 h. Then, the fluorescence was detected by a Multimode SpectraMax plate reader at 37 °C with excitation/emission at 485 nm/535 nm. The background GFP signal of the cells and the media were subtracted, and ROS levels were represented as relative change to shCtrl cells without any treatment.

### Intracellular Fe^2+^ measurement

The intracellular Fe^2+^ levels were measured using the FerroOrange Assay Kit from Dojindo Molecular Technologies (JAPAN) (F374) according to the manufacturer’s instructions. Briefly, MCF-7.TamR shCtrl vs. shLINC00152 cells were seeded on clear bottom, black wall plates, and the next day, treated with 7.5 µM tamoxifen. The FerroOrange dye was added to the media at a 1:1000 dilution immediately after treatment. After 1.5 h, the fluorescence signal was quantified at Ex/Em = 561 nm/600 nm. The signal from only media was subtracted from all measurements, and the relative Fe^2+^ levels were calculated by taking the ratio of tamoxifen-treated shCtrl or shLINC00152 cells to their corresponding untreated counterparts.

### In vivo mice experiments

All the in vivo studies were carried out in accordance with the Institutional Animal Care and Use Committee of the Medical University of South Carolina. Six-to-eight-week-old female Nu/J mice were housed with a temperature-controlled and 12-h light/12-h dark cycle environment. Tumors of MCF-7.Par, TamR, TamR.shCtrl or TamR.shLINC00152 cells were developed by injecting 1 × 10^7^ cells subcutaneously near mammary fat pads. Slow-release estradiol pellets (0.36 mg, 60 days; Innovative Research of America) were implanted subcutaneously (s.c) at the nape of the neck 1 day before injection. Tumor growth was regularly monitored, and size measurements were performed two times per week. Tumor volume was calculated using the formula (length × width^2^)/2. After MCF-7.TamR.shCtrl tumor size reached 100 mm^3^, the mice with shCtrl and shLINC00152 tumors were randomly divided into 2 groups, with 7–8 mice per group. Animals were treated with vehicle (corn oil) or tamoxifen (3 mg/kg in corn oil, by oral gavage) daily. The tumor size of each mouse was recorded twice a week. Mice were sacrificed 28 days after initiation of the treatment. There was no blinding during in vivo experiments. Sample sizes were determined based on previous studies [33, 60].

### Bioinformatics and statistical analysis

The normalized expression matrices of the microarray data sets, GSE6532 [73], GSE58644 [74], GSE42568 [75], GSE87411 [76] and GSE4922 [77] were downloaded from the GEO database, and the TCGA data were downloaded from XenaBrowser (UCSC Genomics Institute) [78] and KM-Plotter database [79]. Selection of datasets were based on availability of the probes for the genes of interest, e.g., LINC00152, the availability of normal tissue in addition to cancerous tissue, the availability of clinical data, such as treatment response and sample collection time, e.g., before and after treatment with endocrine therapy and the survival data. The LINC00152 knockdown (KD) signature was generated using the cumulative list of genes down or upregulated upon LINC00152 knockdown using siLINC00152#1 or siLINC00152#2 in MCF-7.TamR cells. The KD score was then calculated by subtracting the sum of the z-score of the genes downregulated from the sum of the z-scores of the genes upregulated using the SPSS Statistics software. The ferroptosis gene signature comprises genes driving ferroptosis and was obtained from FerrDb V2 database [38]. The Ca^2+^ signaling signature was obtained from the KEGG database [46], and the cAMP signature was generated using genes significantly upregulated (*P*-value < 0.001 and FC > 2) upon treatment of cancer cells with the cAMP analog, 8-CPT-cAMP for 24 h [49]. All ferroptosis, calcium, and cAMP scores were generated by summing up the z scores of the genes in each list separately. The GSEA analyses in low LINC00152 KD score-expressing or low LINC00152-expressing patients were performed using the GSEA tool (Broad Institute).

The results are represented as mean ± standard deviation (SD) or mean ± standard error of the mean (SEM), as indicated in the figure legends. All statistical analyses were performed in GraphPad Prism Software. Comparisons between the two groups were made using unpaired two-sided Student’s *t*-test. Comparison of the tumor volume graphs was done using two-way ANOVA with Dunnett’s multiple comparisons test. Survival curves were generated using the Kaplan–Meier method. Separation of patients was done based on median (GSE6532, GSE42568 and the Hacettepe cohort) or quartile (GSE58644) expression of LINC00152, which are two commonly used patient stratification methods [80]. For survival analysis with the TCGA data, the best cut-off option of KM-Plotter database was utilized. Significance between groups was calculated by the Log-rank test. For correlation analysis, Pearson correlation coefficients were calculated. For chi-square testing of Hacettepe samples, number of patients showing relapse or no relapse within 5-years were compared based on low vs. high LINC00152 expression separated from median. Outlier analysis was performed using Grubbs’ test. Experiments were repeated two to three times independently with similar results.

All other methods, including RNA-seq and data analysis, transient transfection with siRNAs, stable transfections using lentiviral vectors, quantitative RT-PCR analysis, mRNA stability assay using actinomycin D, 3’UTR assay, Western blotting, in situ hybridization and immunohistochemistry are provided in Supplementary Methods. The Supplementary Tables for the sequences of shRNAs (Supplementary Table S3), siRNAs and ASOs (Supplementary Table S4), overexpression vectors (Supplementary Table S5), and the list of antibodies (Supplementary Table S6) are also provided in the Supplementary Information file.

### Supplementary information

Supplementary File

Source Data for Western Blotting

Supplementary Data 1

Supplementary Data 2

## Data Availability

The data that support the findings of this study are available from the corresponding author upon reasonable request. Gene expression data were downloaded from the NCBI Gene Expression Omnibus database under GSE6532, GSE58644, GSE42568, GSE87411, and GSE4922. The RNA-seq data of MCF-7.TamR cells compared to parental were uploaded to the Gene Expression Omnibus database under the BioProject ID: PRJNA1040324. The RNA-seq data of MCF-7.TamR siLINC00152 cells compared to siCtrl were uploaded to the Gene Expression Omnibus database under the BioProject ID: PRJNA1036119. The raw data for western blotting are provided as a source data file.
