# Supplementary material for: Targeting LINC00152 activates cAMP/Ca2+/ferroptosis axis and overcomes tamoxifen resistance in ER+ breast cancer
Source: Cell Death Dis. 2024 Jun 15;15(6):418. doi: 10.1038/s41419-024-06814-3 (PMC11180193; doi:10.1038/s41419-024-06814-3)
Supplement: Supplementary file 1 — Supplementary File [file 41419_2024_6814_MOESM1_ESM.pdf]

SUPPLEMENTARY INFORMATION

Targeting LINC00152 activates cAMP/Ca^{2+}/ferroptosis axis and overcomes tamoxifen resistance in ER+ breast cancer

Ozge Saatci[1,2], Rashedul Alam[1], Kim-Tuyen Huynh-Dam[2], Aynur Isik[3], Meral Uner[3], Nevin Belder[4], Pelin Gulizar Ersan[4], Unal Metin Tokat[4], Burge Ulukan[1], Metin Cetin[1,2], Kubra Calisir[1], Mustafa Emre Gedik[1], Hilal Bal[4], Ozlem Sener Sahin[1], Yasser Riazalhosseini[5,6], Denis Thieffry[7,8], Daniel Gautheret[9], Besim Ogretmen[1], Sercan Aksoy[10], Aysegul Uner[3], Aytekin Akyol[3], Ozgur Sahin[1,2*]

[1]Department of Biochemistry and Molecular Biology, Hollings Cancer Center, Medical University of South Carolina, Charleston, SC, 29425, USA

[2]Department of Drug Discovery and Biomedical Sciences, University of South Carolina, Columbia, SC, 29208, USA

[3]Department of Pathology, Faculty of Medicine, Hacettepe University, 06100, Ankara, TURKEY

[4]Department of Molecular Biology and Genetics, Bilkent University, Ankara, 06800, TURKEY

[5]Department of Human Genetics, McGill University, Montreal, Quebec, CANADA.

[6]Victor Philip Dahdaleh Institute of Genomic Medicine at McGill University, Montreal, Quebec, CANADA.

[7]Département de biologie de l'Ecole normale supérieure, PSL Université, 75005 Paris, FRANCE.

[8]Bioinformatics and Computational Systems Biology of Cancer, U900 Institut Curie - INSERM - Mines ParisTech, PSL Université, 75005 Paris, FRANCE.

[9]Institute for Integrative Biology of the Cell (I2BC), Université Paris-Saclay, CNRS, CEA, 91190, Gif-sur-Yvette, FRANCE.

[10]Department of Medical Oncology, Hacettepe University Cancer Institute, 06100, Ankara, TURKEY

Keywords: ER+ breast cancer/tamoxifen resistance/lncRNA/calcium/cAMP/ferroptosis

***Corresponding author**

Ozgur Sahin, PhD

Professor and SmartState Endowed Chair

Department of Biochemistry and Molecular Biology

Hollings Cancer Center

Medical University of South Carolina

86 Jonathan Lucas Street, Room HO712F,

Charleston, SC 29425

Phone: +1-843-792-0166

E-mail: sahinozgur@gmail.com or sahin@musc.edu

SUPPLEMENTARY METHODS

RNA-sequencing (RNA-seq) and data analysis

RNA-seq of parental vs. TamR cells

Ribosomal RNA (rRNA)-depleted libraries were generated for each sample, and these sequences were multiplexed. RNA seq was performed for each condition (MCF-7.Par and MCF-7.TamR) in triplicates using the Illumina HiSeq 2000 platform at McGill University and Genome Quebec Innovation Centre. Around 65 million paired-end 2×100bp reads were generated for each replicate. FASTQC tool was used to check the quality of the sequencing data. Raw FASTQ sequences were aligned to the UCSC human reference genome (hg19) using TopHat v2.1.0 with default parameters. *Cufflinks* was used to assemble and quantify transcripts from the mapped sequences. By utilizing reference genome annotation (UCSC hg19), *Cuffmerge* produced a single merged reference transcripts for differential expression analysis. For the identification of the differentially expressed genes, *Cuffdiff* was used with the default parameters. The 330 differentially expressed lncRNAs between parental vs. TamR cells (q value = 0.05) were retrieved and sorted based on the abundance (FPKM) in TamR cells (**Supplementary Data 2**).

RNA-seq of TamR cells with LINC00152 knockdown

The RNA-seq of MCF-7.TamR siControl, siLINC00152#1 and siLINC00152#2 was performed for each condition in duplicates at University of South Carolina Functional Genomics Core. FASTQC tool was used to check the quality of the sequencing data. Raw FASTQ sequences were aligned to the UCSC human reference genome (hg38) using TopHat v2.1.0 with default parameters. *Cufflinks* was used to assemble and quantify transcripts from the mapped sequences. *Cuffmerge* produced a single merged reference transcripts for differential expression analysis. For the identification of the differentially expressed genes, *Cuffdiff* was used with the default parameters, and the genes differentially expressed upon LINC00152 knockdown (siControl vs. siLINC00152#1 and/or siControl vs. siLINC00152#2) with the cut-off of $0.67 > FC > 1.5$ and $P < 0.1$ were determined. Principal component analysis (PCA) was done with unnormalized and filtered (removal of lowly expressed genes) count data. Volcano plot was drawn with differentially expressed, genes

and up- and down -regulated genes were highlighted (abs(logFC)>1 & p-value<0.05). For these plots, the ggplot2 package was used in R version 4.3.2. Gene Set Enrichment Analysis (GSEA) was done with GSEA software version 4.3.2. with the whole unfiltered count list.

Quantitative RT-PCR analysis

Total RNA was extracted from cultured cells using TRIsure (Bioline), and cDNAs were generated using RevertAid RT Reverse Transcription Kit (Life Technologies). qRT-PCR analysis was performed with gene-specific primers using LightCycler 480 SYBR Green I Master kit (Roche). *HPRT1* and *ACTB* were used as housekeeping genes. The average Ct value was calculated from triplicates of each sample, and the relative mRNA expression was determined. Sequences of the qRT-PCR primers are listed in **Supplementary Table S2**.

Stable transfections using lentiviral vectors

An shRNA specifically targeting LINC00152 was designed using Vector Builder. The sequences are provided in **Supplementary Table S3**. To generate viral particles carrying shRNA vector, 6 μg of vectors along with the psPAX2 and pMD2.G packaging plasmids were co-transfected into HEK293FT cells in 6-well plate using lipofectamine (Invitrogen). After 48 hours of incubation, the viral particles were collected and transduced into MCF-7.TamR cells. Further selection was done by treating cells with the medium containing 2 μg/mL puromycin for 10 days. The PDE4D ORF expressing MCF-7.TamR.shLINC cells were generated by transfecting the MCF-7.TamR.shLINC cells with a PDE4D ORF vector (PDE4D cDNA (NM_006203.4) from GenScript), followed by selection with geneticin.

Transient transfection with siRNAs, ASOs, and overexpression vectors

Transfections were done in P/S-free growth medium with reduced serum using siRNAs at a concentration of 40 nM, ASOs at a concentration of 80 nM, overexpression vectors at a concentration of 200 ng for LINC00152 (cloned from TamR cells into pCDNA3.1) and 500 ng for GPX4 (GenScript), xCT (Adgene) and TRPC1 (Addgene), using Lipofectamine 2000^{TM} (Invitrogen) as previously described (1). The list of siRNAs, ASOs and overexpression vectors used are provided in **Supplementary Table S4 and S5**.

mRNA stability assay using Actinomycin D

To measure the changes in the stability of PDE4D mRNA upon LINC00152 knockdown, the TamR cells were transfected with 40 nM of siCtrl and siLINC00152. Two days later, cells were treated with 5 ug/mL actinomycin D and collected after 0, 60, 120, 240 minutes. RNA was extracted using TRIsure (Bioline), and cDNAs were generated using RevertAid RT Reverse Transcription Kit (Life Technologies). qRT-PCR analysis was performed using LightCycler 480 SYBR Green I Master kit (Roche). The fold change of PDE4D mRNA was measured by normalizing each time point to time zero for siCtrl and siLINC separately.

3'UTR assay

To test the binding of LINC00152 to *PDE4D* 3'UTR, cells were co-transfected with the LINC00152 overexpression vector together with *PDE4D* 3'UTR cloned in 3 different vectors. 48 hours after transfection, cells were lysed, and luminescence signal was quantified using the Luciferase Assay System (Promega).

Western blotting

Western blotting was done as describe previously (2-4). Briefly, total protein was extracted using RIPA buffer (150 mmol/L NaCl, 50 mmol/L Tris base pH 8.0, 1 mmol/L EDTA, 0.5% sodium deoxycholate, 1% NP40, 0.1% SDS, 1 mmol/L DTT, and 1 mmol/L Na3VO4) supplemented with Complete Protease Inhibitor (Roche). Protein concentrations were measured using BCA Protein Assay (Thermo Scientific). Equal amounts of protein lysates (15-20 μg) were separated on a 10% SDS-PAGE, transferred onto polyvinylidene difluoride membrane (Bio-Rad) and incubated with primary antibodies listed in **Supplementary Table S6**. The blots were developed using enhanced chemiluminescence (ECL) detection kit (Amersham Biosciences) after incubation with horseradish peroxidase–conjugated secondary antibody. β-Actin was used as a loading control.

In situ hybridization and Immunohistochemistry

LINC00152 RNA in situ hybridization was performed manually by RNAscope™ Probe- Hs-LINC00152 (Cat No. 464401, Advanced Cell Diagnostics (ACD), Hayward, CA) and RNAscope® 2.5 HD Detection Reagent-BROWN kit (Cat. No: 322310, ACD) in formalin-fixed paraffin-embedded (FFPE) tissues as defined in vendor's instructions. Briefly, 4-micron-thick unstained sections from FFPE blocks were

prepared, and deparaffinization with xylene and hydration with graded ethanol washes (96%, 90%, and 70%, respectively) was done. After endogenous peroxidase blockage, signal amplification and background suppression were performed as recommended in manual. Signals were developed with DAB and haematoxylin was used for counterstaining.

For the IHC staining of PDE4D protein in patient tumor samples from the Hacettepe cohort, antigen retrieval was done for 10 min with the EDTA buffer, and slides were incubated with the PDE4D antibody (Proteintech, clone 12918) at a dilution of 1:100 for 20 minutes at room temperature using Leica BOND-MAX/ISH automated immune-stainer.

The IHC staining of PDE4D in the FFPE slides from xenograft tumors was performed by deparaffinization at 60 ^{o}C for 1 hour, followed by rehydration in citrisolv for 5 min (3 times), 100% ethanol for 5 min (twice), 95% ethanol for 5 min (twice), deionized water for 5 min (twice). Antigen retrieval was done with Tris-EDTA pH=9 at 96 ^{o}C for 15 min, followed by cooling down to room temperature for 30 min, and washing with TBST for 5 min. Blocking was done at room temperature with Buffer W (IBA-Lifesciences) for 30 min. Then, the slides were incubated with the PDE4D antibody (Proteintech, clone 12918) at a dilution of 1:100 for 20 minutes at room temperature.

SUPPLEMENTARY FIGURES

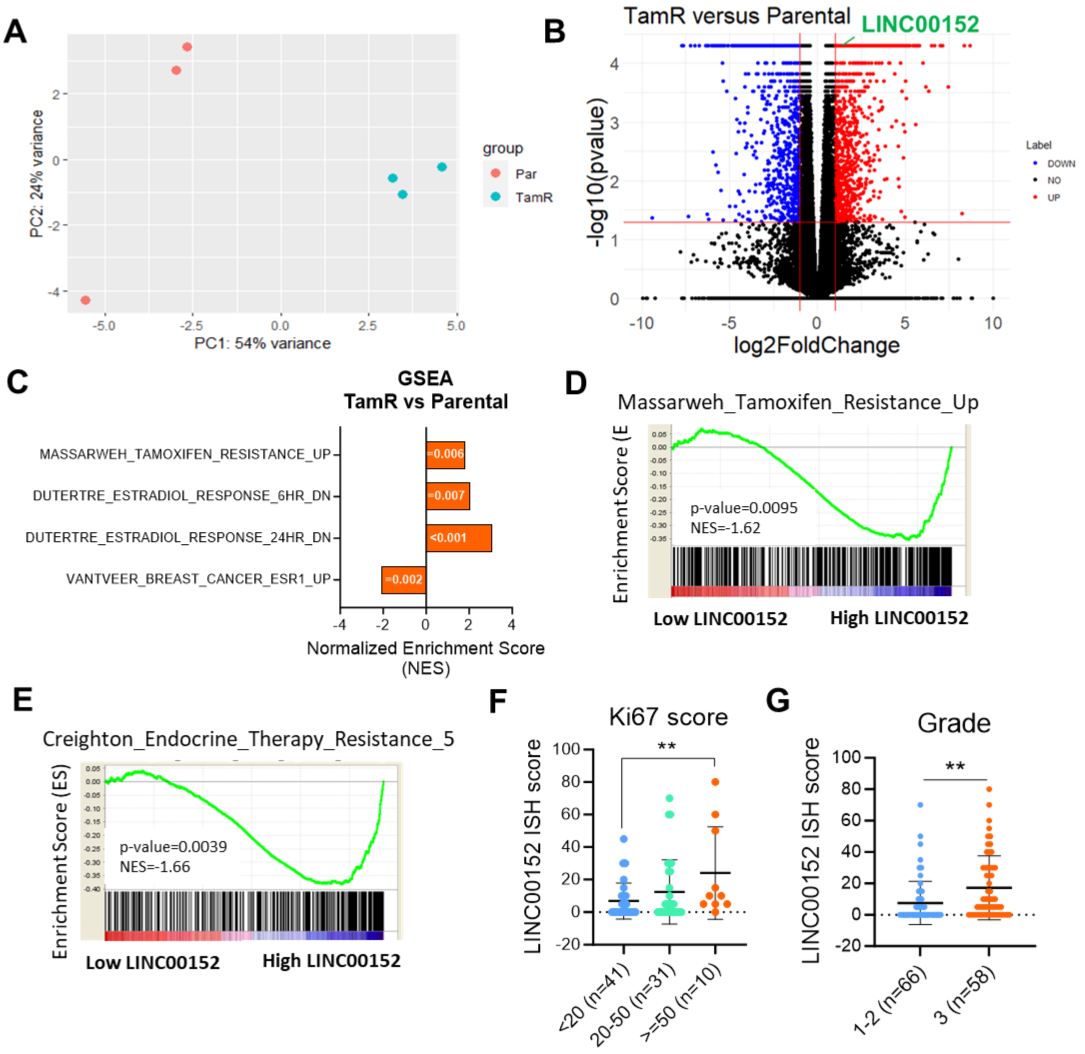

Supplementary Figure 1. Higher LINC00152 expression correlates with tamoxifen resistance, and higher proliferation and grade of ER+ patients treated with tamoxifen. **A** PCA plot showing the sample and replicate clustering in RNA-seq analysis. **B** Volcano plot showing significantly differentially expressed genes in TamR vs. parental cells where LINC00152 is highlighted (log2FC>1 & *P*-value<0.05). **C** GSEA analysis showing gene sets related to tamoxifen resistance and estrogen response enriched in MCF-7.TamR or MCF-7.Par cells. **D, E** GSEA analysis of genes upregulated in tamoxifen (D) or endocrine (E) resistance showing enrichment in patients expressing high LINC00152. **F** The LINC00152 ISH score

in ER+ breast cancer patients from the Hacettepe cohort with low (Ki67 score < 20), moderate (20 < Ki67 score < 50) and highly (Ki67 score > 50) proliferative tumors. **G** The LINC00152 ISH score in ER+ breast cancer patients from the Hacettepe cohort with low (1-2) vs. high (3) grade tumors. Data are presented as mean values ± standard deviation (SD). *P*-value for C-E was generated using the GSEA software (Broad Institute) and *P*-values for F and G were calculated with the unpaired, two-tailed Student's t-test. ** $P<0.01$. NES: normalized enrichment score.

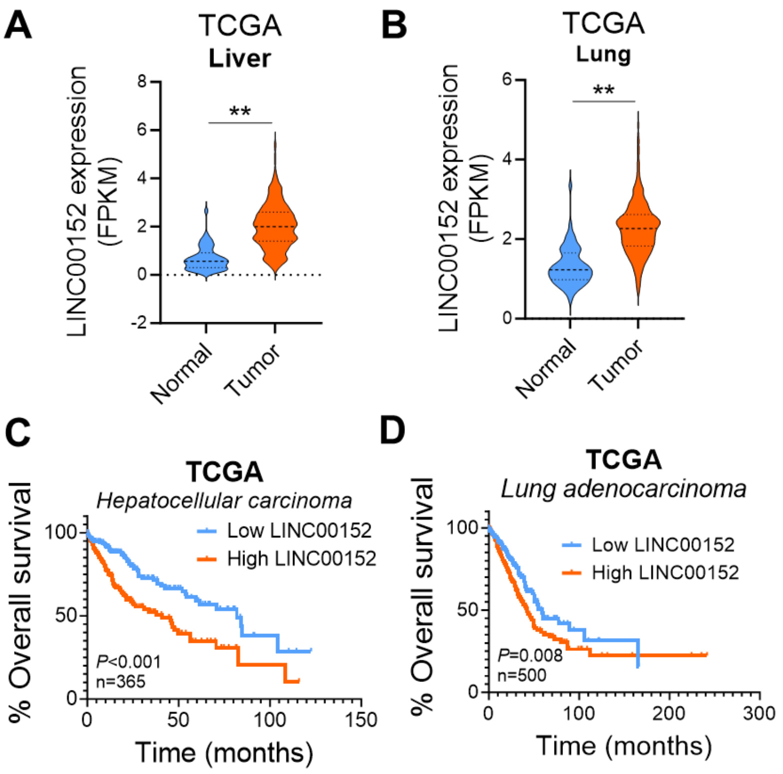

Supplementary Figure 2. LINC00152 is upregulated and associated with worse survival in cancers other than breast. A LINC00152 expression in normal liver vs. hepatocellular carcinoma. **B** LINC00152 mRNA expression in normal lung vs. lung adenocarcinoma. **C, D** Kaplan Meier overall survival analysis in hepatocellular carcinoma (C) and lung adenocarcinoma (D) patients based on LINC00152 expression. Data are presented as mean values ± standard deviation (SD). *P*-values were calculated with unpaired, two-tailed Student's t-test or with Log-Rank test. ** *P*<0.01.

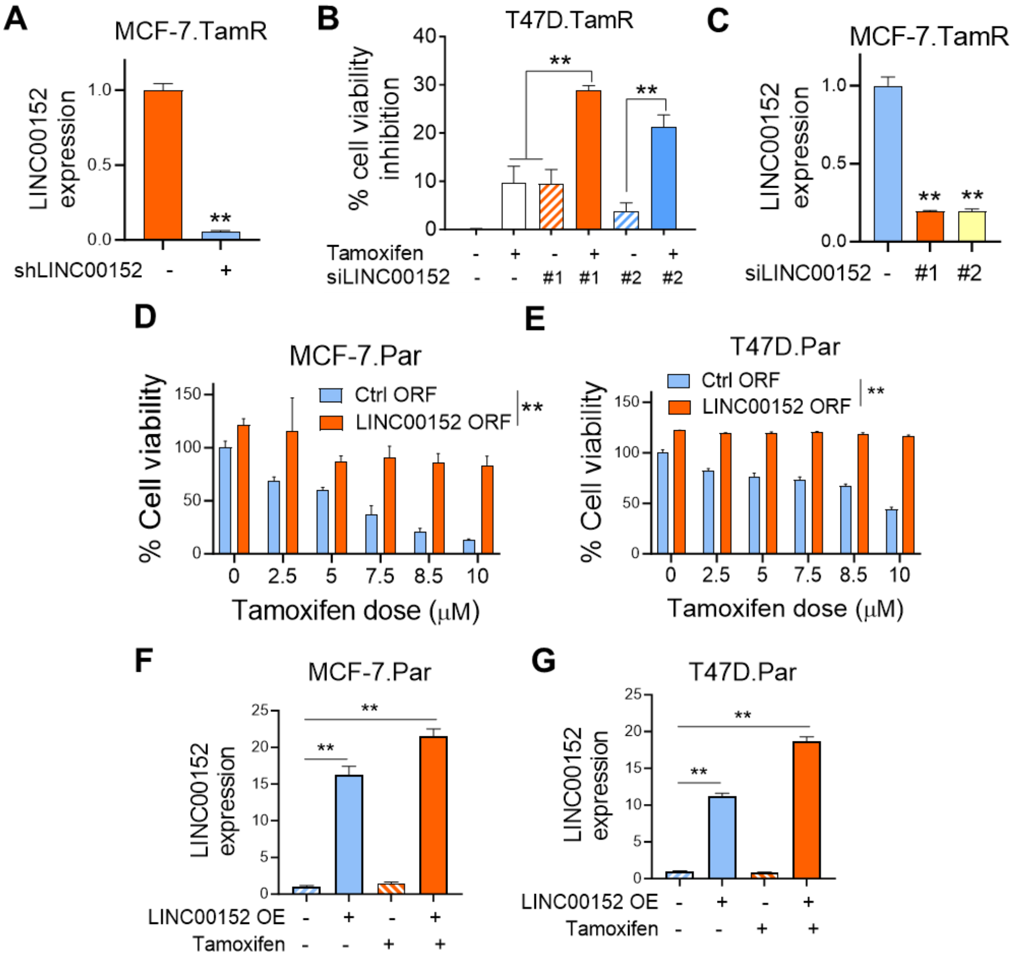

Supplementary Figure 3. The effects of LINC00152 knockdown or overexpression on tamoxifen sensitivity/resistance. A LINC00152 knockdown efficacy in shLINC00152-expressing MCF-7.TamR cells. **B** Percent cell viability inhibition in siLINC00152-transfected T47D.TamR cells treated with tamoxifen. **C** qRT-PCR analysis of LINC00152 in siLINC00152-transfected MCF-7.TamR cells transfected with two different siRNAs. **D, E** Percent cell viability in LINC00152-overexpressing MCF-7.Par (D) and T47D.Par (E) cells under tamoxifen treatment. **F, G** qRT-PCR analysis of LINC00152 in MCF-7.Par (F) and T47D.Par (G) cells overexpressing LINC00152 and treated with or without tamoxifen. Data are presented as mean values ± standard deviation (SD). *P*-values were calculated with the unpaired or paired two-tailed Student's t-test. ** $P < 0.01$.

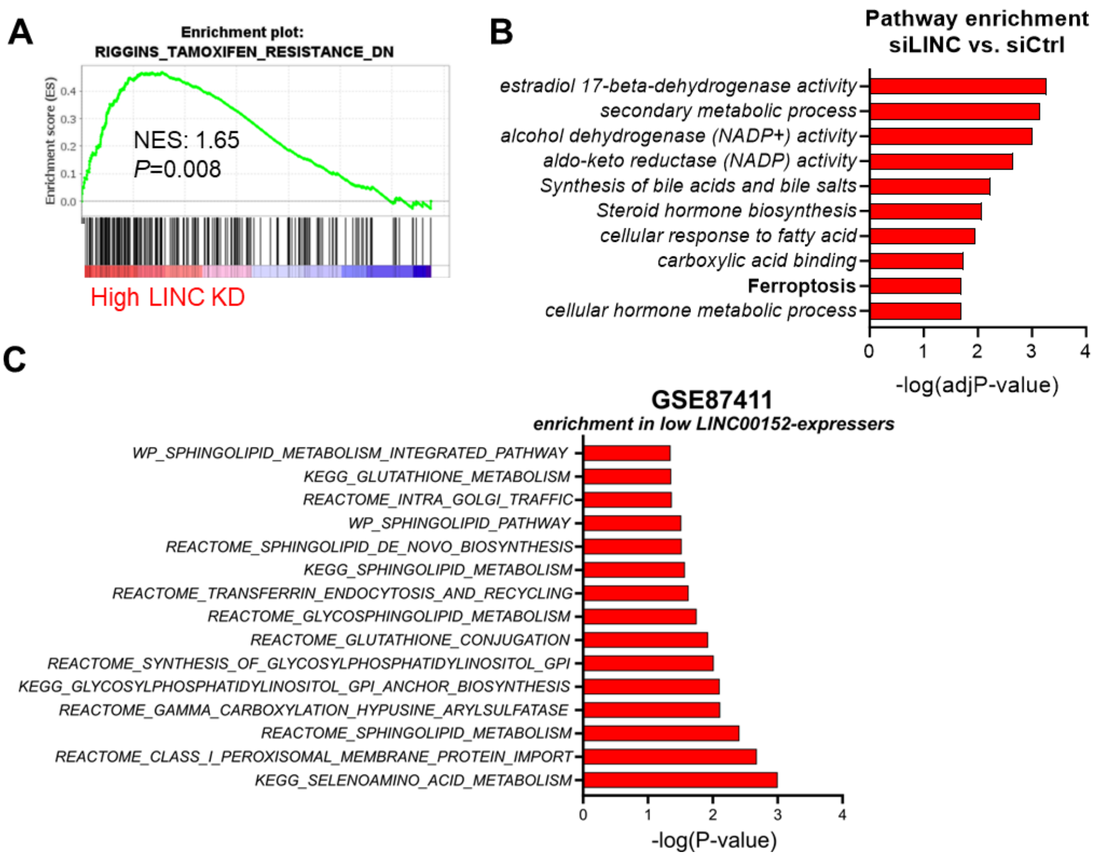

Supplementary Figure 4. Clinical association of LINC00152 with tamoxifen resistance and ferroptosis. A GSEA analysis in ER+ breast cancer patients showing enrichment of genes downregulated in tamoxifen resistance among patients expressing high LINC00152 knockdown (KD) score. **B** Pathway enrichment analysis among the differentially expressed genes in siLINC00152-transfected MCF-7.TamR cells. **C** GSEA analysis showing the gene sets enriched in low LINC00152-expressing ER+ breast cancer patients treated with endocrine therapy from GSE87411.

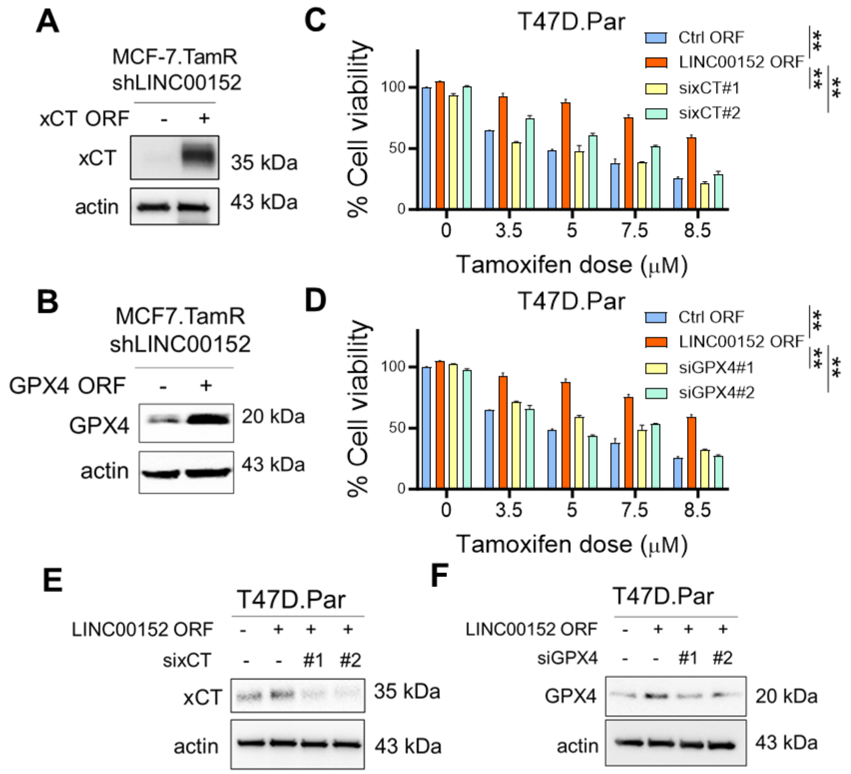

Supplementary Figure 5. The roles of xCT and GPX4 in LINC00152-mediated tamoxifen resistance. **A**, **B** Western blot analysis of xCT (A) and GPX4 (B) upon their overexpression in shLINC00152-expressing MCF-7.TamR cells. **C, D** Percentage cell viability inhibition in T47D.Par cells transfected with LINC00152 ORF with or without siRNAs against xCT (C) or GPX4 (D). **E, F** Western blot analysis of xCT (E) and GPX4 (F) from C and D. Data are presented as mean values ± standard deviation (SD). *P*-values were calculated with the paired, two-tailed Student's t-test. * $P < 0.05$; ** $P < 0.01$.

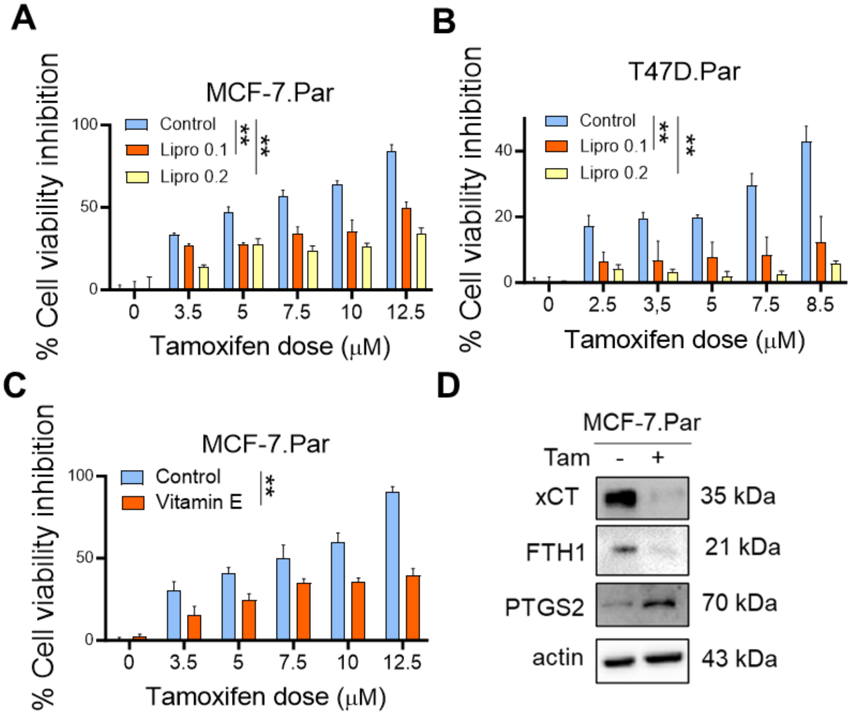

Supplementary Figure 6. Ferroptosis is a mechanism of tamoxifen sensitivity in ER+ breast cancer cells. A, **B** Percentage cell viability inhibition in MCF-7.Par (A) and T47D.Par (B) cells treated with tamoxifen with or without 0.1 or 0.2 μM of the ferroptosis inhibitor, liproxstatin-1. **C** Percentage cell viability inhibition in MCF-7.Par cells treated with tamoxifen with or without 10 μM Vitamin E. **D** Western blot analysis of ferroptosis markers in tamoxifen-treated MCF-7.Par cells. Data are presented as mean values ± standard deviation (SD). *P*-values were calculated with the paired, two-tailed Student's t-test. ** $P < 0.01$.

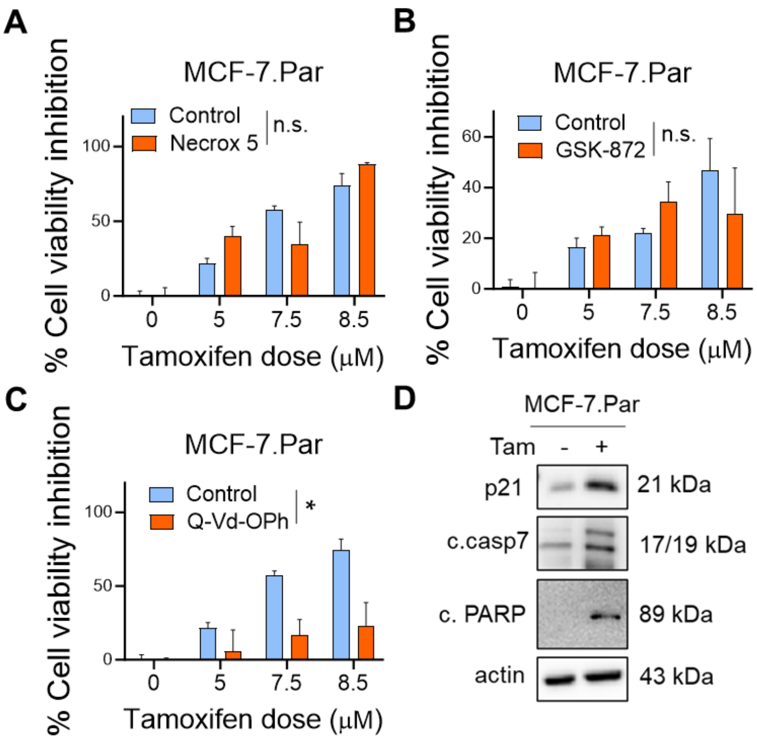

Supplementary Figure 7. Involvement of other cell death mechanisms to tamoxifen sensitivity. A-C Percent cell viability inhibition in MCF-7.Par cells treated with tamoxifen with or without Necrox-5 (A), GSK-872 (B) or Q-Vd-OPh (C). **D** Western blot of apoptosis markers in tamoxifen treated MCF-7.Par cells for 48 hrs. Data are presented as mean values ± standard deviation (SD). *P*-values were calculated with the paired, two-tailed Student's t-test. * $P < 0.05$; n.s. not significant.

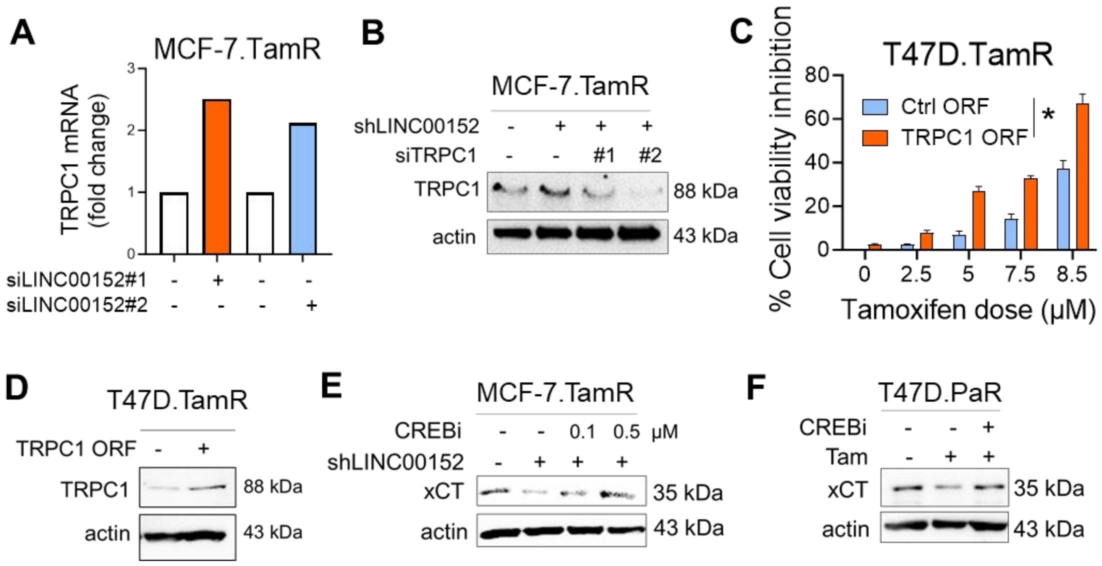

Supplementary Figure 8. The roles of TRPC1 and CREB in LINC00152 inhibition-mediated tamoxifen sensitization. A TRPC1 fold change in MCF-7.TamR cells transfected with two different siRNAs against LINC00152 as determined by RNA-seq. **B** Western blot of TRPC1 in siTRPC1-expressing MCF7.TamR.shLINC00152 cells. **C** Percentage cell viability inhibition in T47D.TamR cells transfected with TRPC1 ORF and treated with tamoxifen. **D** Western blot of TRPC1 in TRPC1-overexpressing T47D.TamR cells. **E** Western blot of xCT in shLINC00152-expressing cells treated with CREB inhibitor (666-15). **F** Western blot of xCT in T47D.Par cells treated with tamoxifen with or without 0.1 μM CREB inhibitor. Data are presented as mean values ± standard deviation (SD). *P*-values were calculated with the paired, two-tailed Student's t-test. * $P < 0.05$.

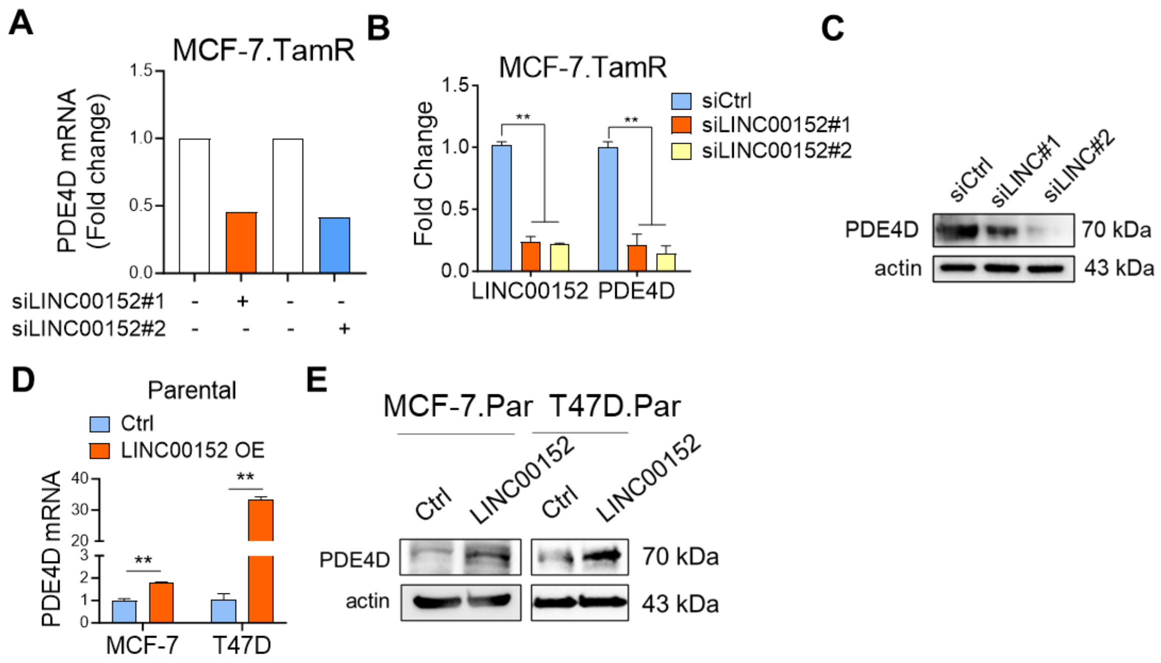

Supplementary Fig 9. LINC00152 inhibition reduces PDE4D while its overexpression induces it. A PDE4D fold change in MCF-7.TamR cells transfected with two different siRNAs against LINC00152 as determined by RNA-seq. **B** qRT-PCR analysis of LINC00152 and PDE4D in MCF-7.TamR cells transfected with two different siRNAs against LINC00152. **C** Western blot analysis of PDE4D in MCF-7.TamR cells transfected with two different siRNAs against LINC00152. **D, E** qRT-PCR (D) and Western blot (E) analysis of PDE4D in parental MCF-7 and T47D cells overexpressing LINC00152. Data are presented as mean values ± standard deviation (SD). *P*-values were calculated with the unpaired, two-tailed Student's t-test. ** $P < 0.01$.

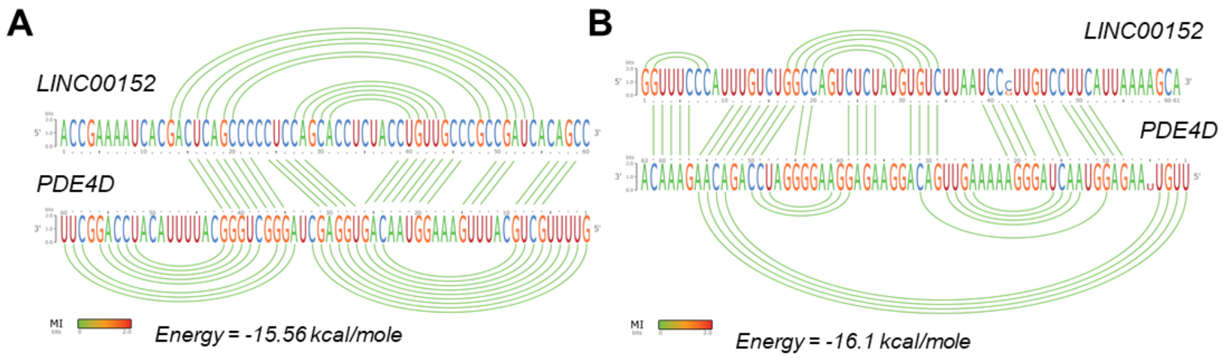

Supplementary Figure 10. Predicted interactions between LINC00152 and *PDE4D* 3'UTR. A, B Two of the binding sites between LINC00152 and *PDE4D* 3'UTR as predicted by IntaRNA database. The visualization of the interaction was obtained using RILogo (https://rth.dk/resources/rilogo/). No mutual interaction (MI) between the bases has been detected. The predicted binding energies are provided.

SUPPLEMENTARY TABLES

Supplementary Data 1. Comprehensive GSEA analysis among the differentially expressed genes in MCF-7.TamR cells compared to parental counterparts.

Supplementary Data 1 is provided as a separate excel file.

Supplementary Data 2. Significantly differentially expressed lncRNAs in MCF-7.TamR cells compared to parental counterpart.

Supplementary Data 2 is provided as a separate excel file.

Supplementary Table S1. The sequence of LINC00152 probe for RNA pull-down.

Gene Name	NCBI Gene ID	Probe sequence
LINC00152	112597	5'-CACCTTCCCAGGAACTGTGCTGTGAAGATCTGAAGACAGGC-3'

Supplementary Table S2. The sequence of qRT-PCR primers.

Gene Name	NCBI Gene ID		Primer sequence
ACTB	60	Forward	5'-CCAACCGCGAGAAGATGA-3'
		Reverse	5'-CCAGAGGCGTACAGGGATAG-3'
HPRT	3251	Forward	5'-TGACCTTGATTTATTTTGCATACC-3'
		Reverse	5'-CGAGCAAGACGTTCAGTCCT-3'
PDE4D	1612	Forward	5'-ATTGCCAGTGATATACACGGA-3'
		Reverse	5'-GTTTCGTACAGTTCGCAGAC-3'
LINC00152	112597	Forward	5'-GAGCCACCAGCCTCTCCTTG-3'
		Reverse	5'-GGGCTGAGTCGTGATTTTCG-3'

Supplementary Table S3. The sequence of shRNA against LINC00152.

Gene Name	NCBI Gene ID	Company	Vector Name	Sequence
LINC00152	112597	Vector Builder	pLV[shRNA]-EGFP:T2A:Puro-U6>(sh-CYTOR-4)	CAGGAAGCTCTATGACACATTCAAGAG ATGTGT CATAGAGCTTCCTG

Supplementary Table S4. Sequences of siRNAs and ASOs.

Gene Name	NCBI Gene ID	Company	Catalog number	Sequence
LINC00152#1	112597	Dharmacon	N-015149-20-005	GUCCUUCAUUAAAAGCAAA
LINC00152#2	112597	Dharmacon	N-015149-18-005	ACAGGAAGCUCUAUGACAC
TRPC1#1	7220	Dharmacon	D-004191-04	GAGAAGAACUGCAGUCCUU
TRPC1#2	7220	Dharmacon	D-004191-05	UCAGGUGACUUGAACAUAA
GPX4#1	2879	Dharmacon	D-011676-17	GCUGCGUGGUGAAGCGCUA
GPX4#2	2879	Dharmacon	D-011676-4	CGUCAAAUUCGAUAUGUUC
xCT#1	23657	Dharmacon	D-007612-01	GGAAGUCUUUGGUCCAUUA
xCT#2	23657	Dharmacon	D-007612-02	GGAGUUAUGCAGCUAAUUA
ASO#1	112597	IDT	CYTOR:PDE4D 2MOE SBO 21mer #1	TCAAGTGTGTCATAGAGCTTC
ASO#2	112597	IDT	CYTOR:PDE4D 2MOE SBO 22mer #2	GTCTGTCATATTCGATCAAGTG

Supplementary Table S5. List of overexpression vectors used.

Gene Name	NCBI Gene ID	Company	Vector Name
GPX4	2879	GenScript	GPX4, transcript variant 1, mRNA. Accession No.:NM_002085.5
xCT	23657	Addgene	pLexM-SLC7A11
TRPC1	7220	Addgene	TRPC1-FLAG

Supplementary Table S6. List of antibodies used in Western blot (WB) experiments.

Antibody	Provider	Catalog number	WB dilution
Beta-actin	MP Biomedicals	691001	1:10000
PDE4D	ProteinTech	12918-1-AP	1:1000
GPX4	Cell Signaling	52455	1:1000
xCT	Cell Signaling	12691	1:1000
FTH1	Cell Signaling	4393	1:1000
p-PKA (Thr197)	Cell Signaling	4781	1:1000
p-CREB (S133)	Cell Signaling	9198	1:1000
PKA	Cell Signaling	5842	1:1000
CREB	Cell Signaling	4820	1:1000
TRPC1	Santa Cruz	sc-133076	1:1000
PTGS2	SantaCruz	sc-19999	1:1000
P21	BD	556430	1:1000
Cleaved caspase 7	Cell Signaling	5625	1:1000
Cleaved PARP	Cell Signaling	8438	1:1000

SUPPLEMENTARY REFERENCES

1. Assidicky R, Tokat UM, Tarman IO, Saatci O, Ersan PG, Raza U, et al. Targeting HIF1-alpha/miR-326/ITGA5 axis potentiates chemotherapy response in triple-negative breast cancer. Breast Cancer Res Treat. 2022;193(2):331-348.

2. Akbulut O, Lengerli D, Saatci O, Duman E, Seker UOS, Isik A, et al. A Highly Potent TACC3 Inhibitor as a Novel Anticancer Drug Candidate. Mol Cancer Ther. 2020;19(6):1243-54.

3. Saatci O, Akbulut O, Cetin M, Sikirzhytski V, Uner M, Lengerli D, et al. Targeting TACC3 represents a novel vulnerability in highly aggressive breast cancers with centrosome amplification. Cell Death Differ. 2023;30(5):1305-19.

4. Saatci O, Kaymak A, Raza U, Ersan PG, Akbulut O, Banister CE, et al. Targeting lysyl oxidase (LOX) overcomes chemotherapy resistance in triple negative breast cancer. Nat Commun. 2020;11(1):2416.
