# Supplementary material for: Targeting LINC00152 activates cAMP/Ca2+/ferroptosis axis and overcomes tamoxifen resistance in ER+ breast cancer
Source: Cell Death Dis. 2024 Jun 15;15(6):418. doi: 10.1038/s41419-024-06814-3 (PMC11180193; doi:10.1038/s41419-024-06814-3)

Fig. 2K

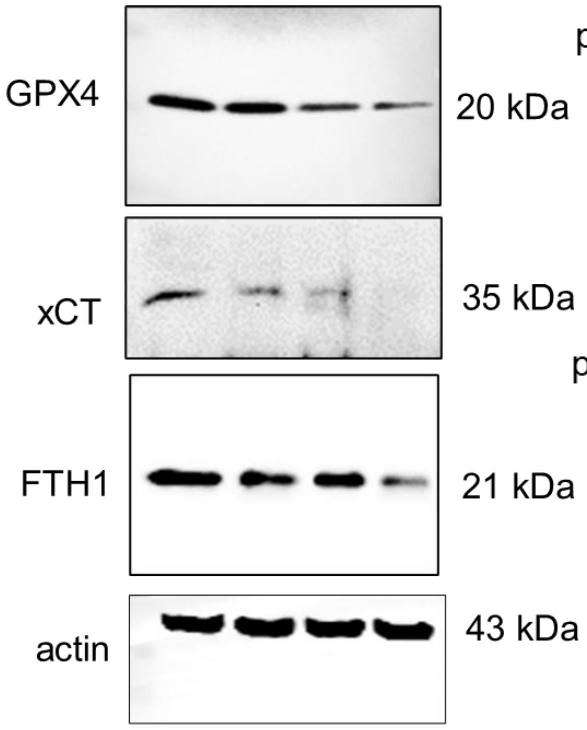

Fig. 3I

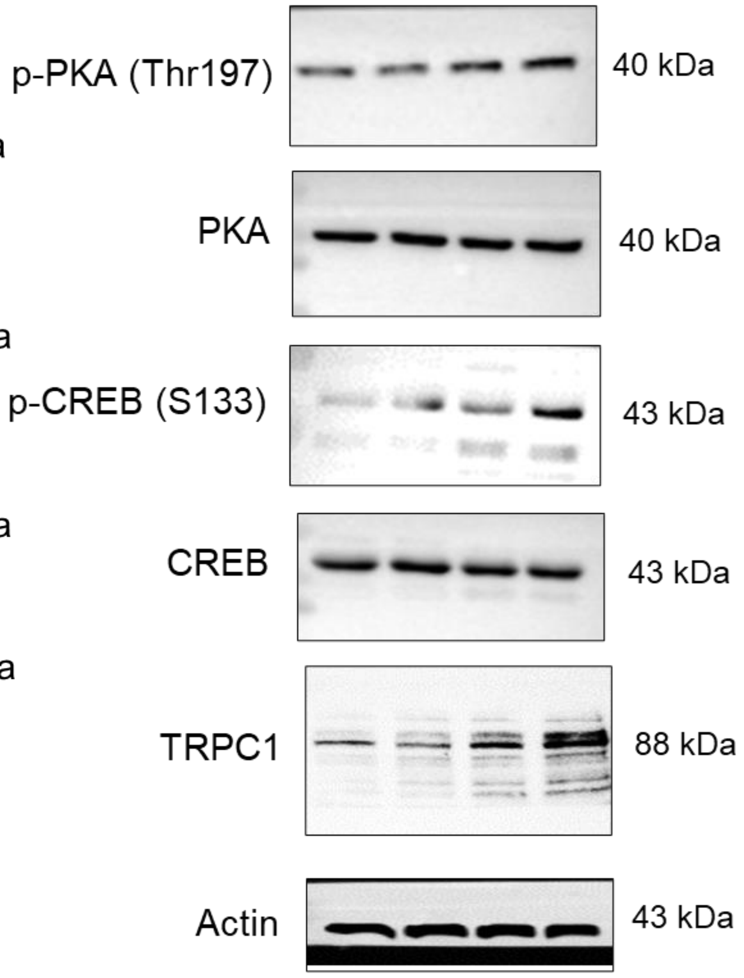

Fig. 4B

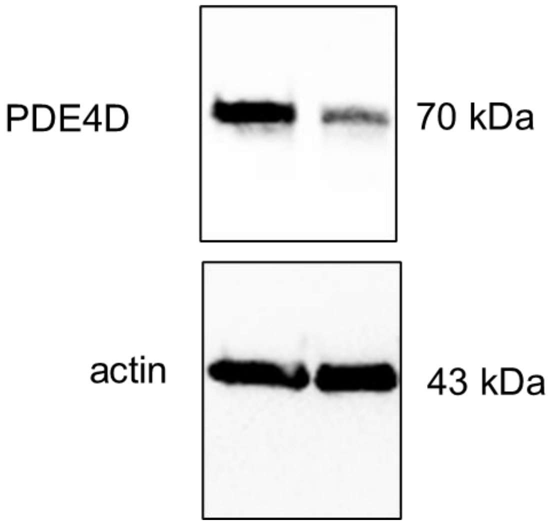

Fig. 4C

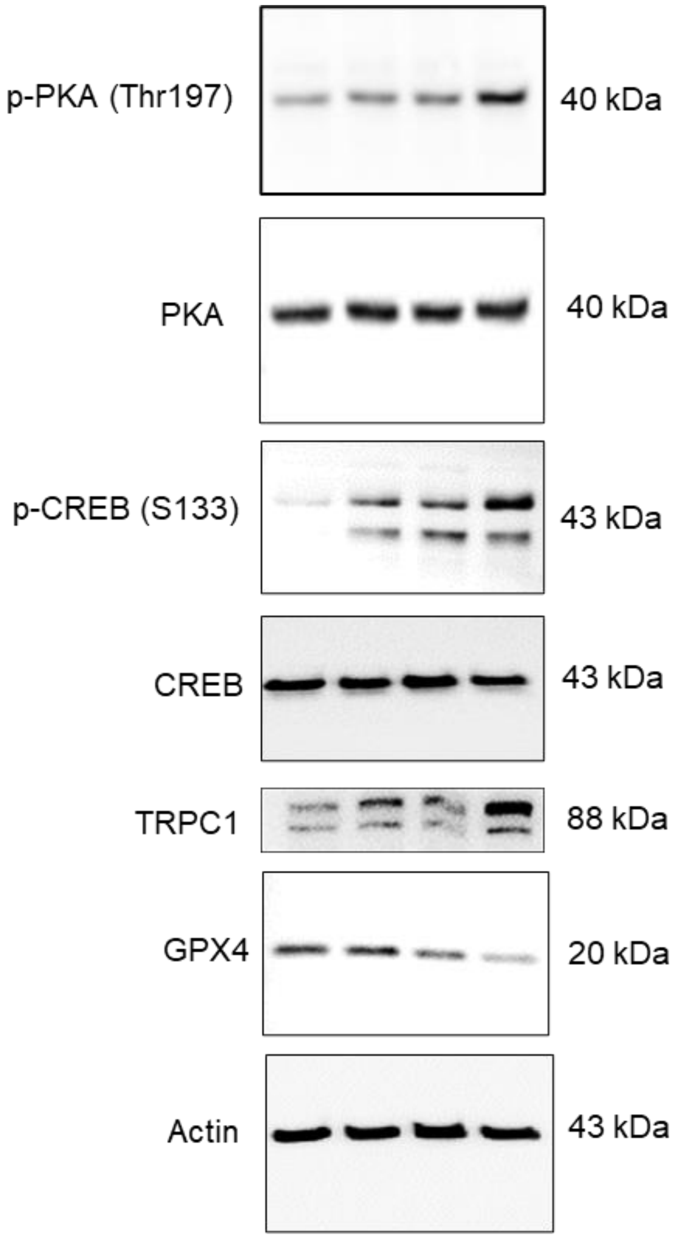

Fig. 4H

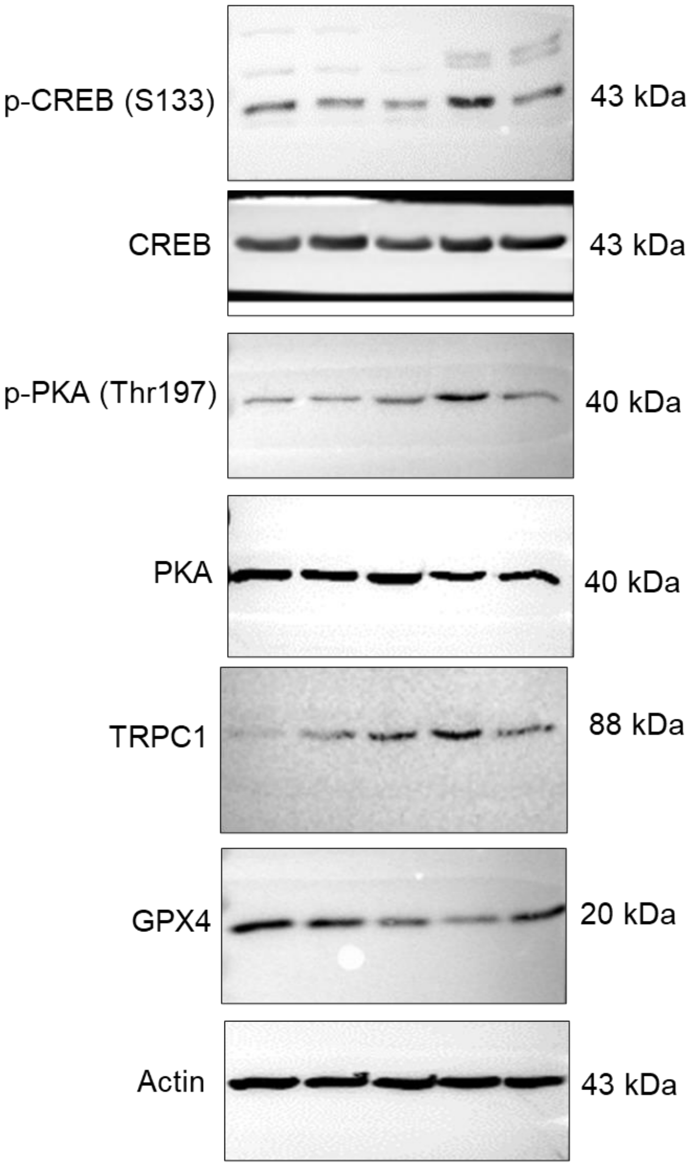

Fig. S5A

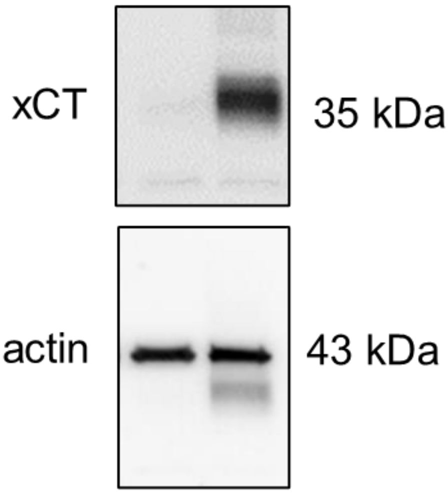

Fig. S5B

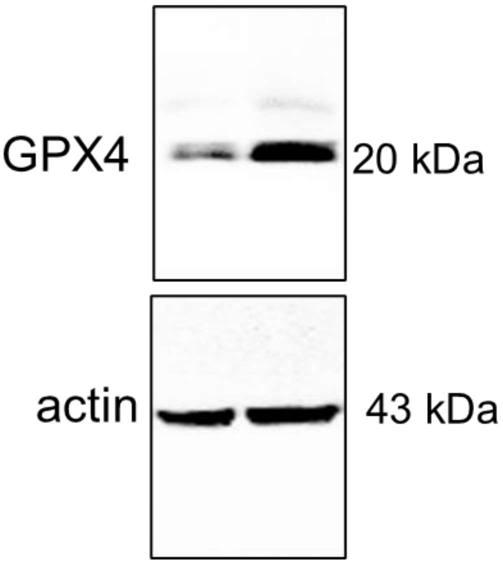

Fig. S5E

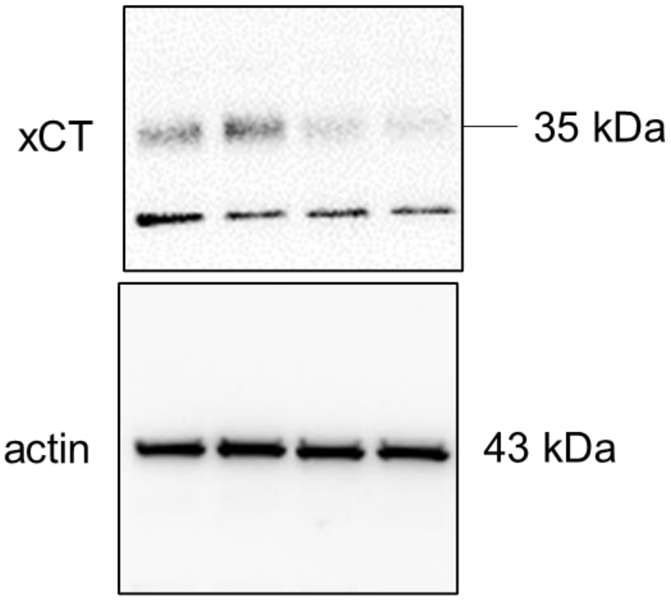

Fig. S5F

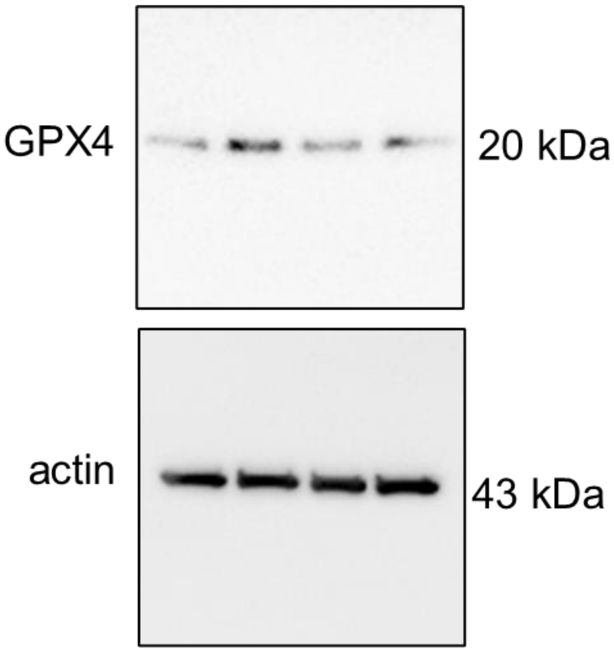

Fig. S6D

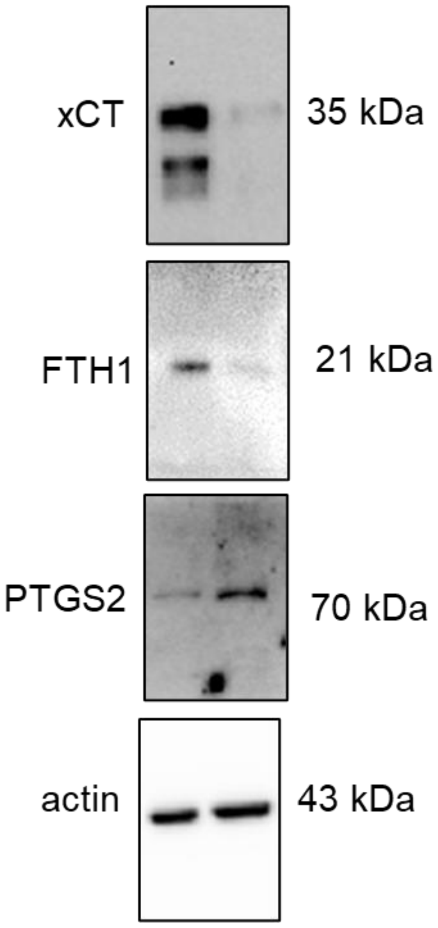

Fig. S7D

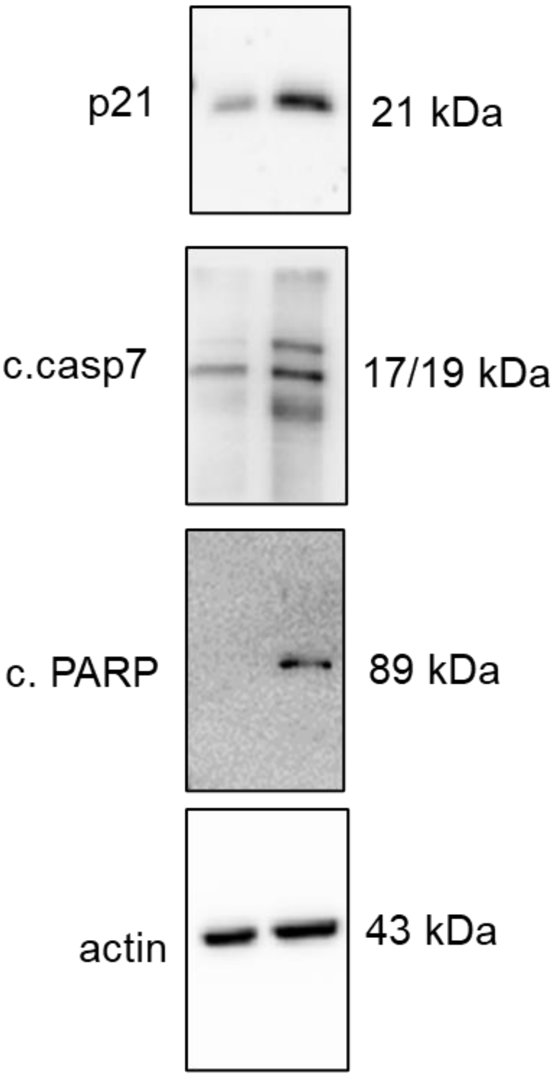

Fig. S8B

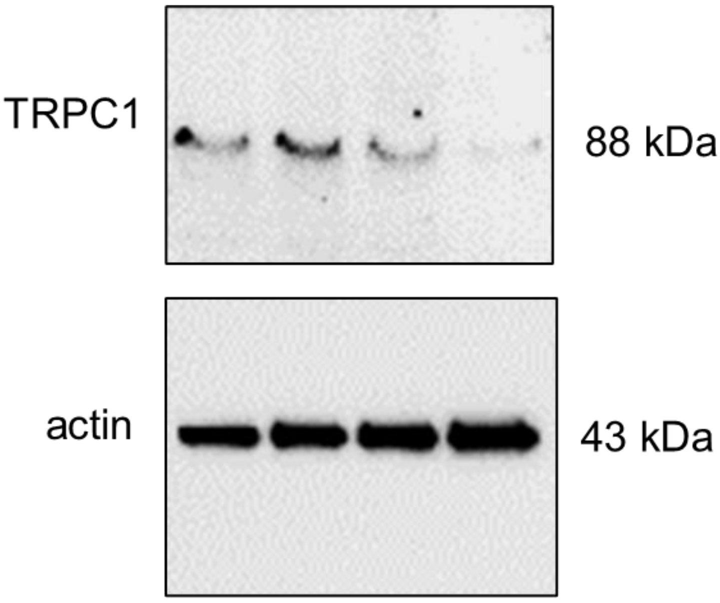

Fig. S8D

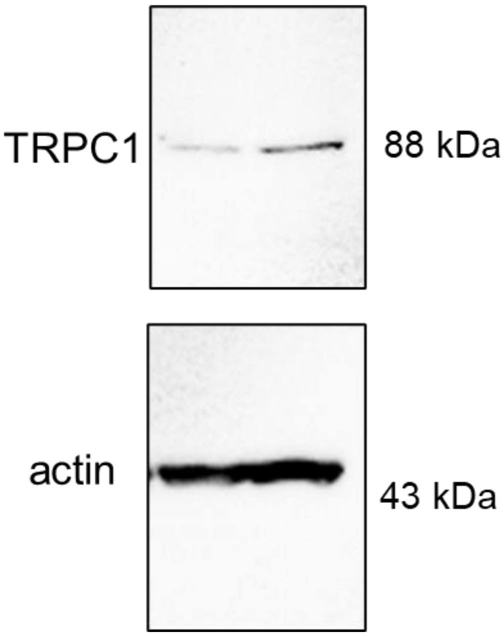

Fig. S8E

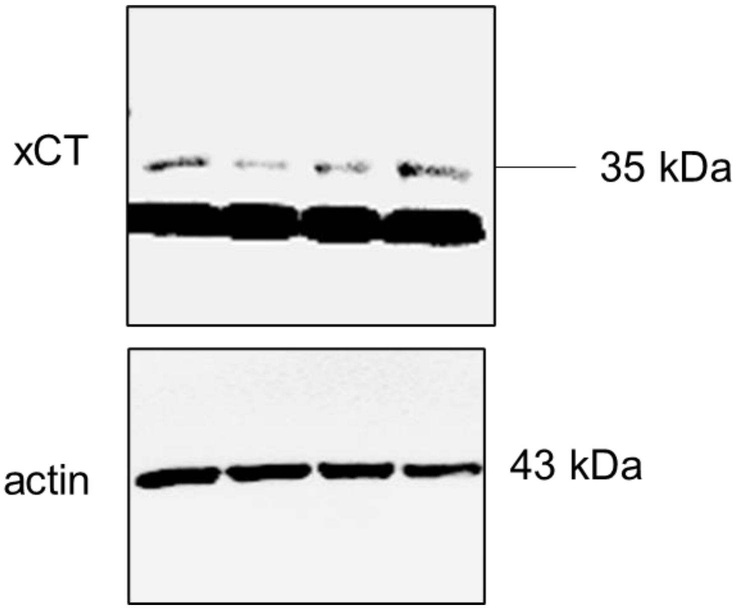

Fig. S8F

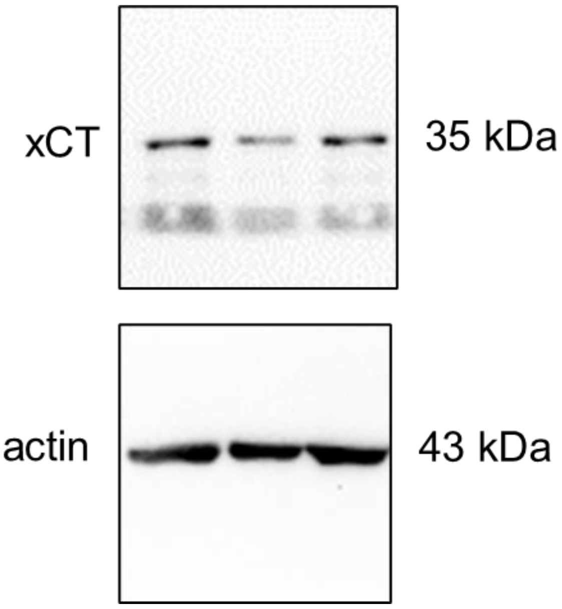

Fig. S9C

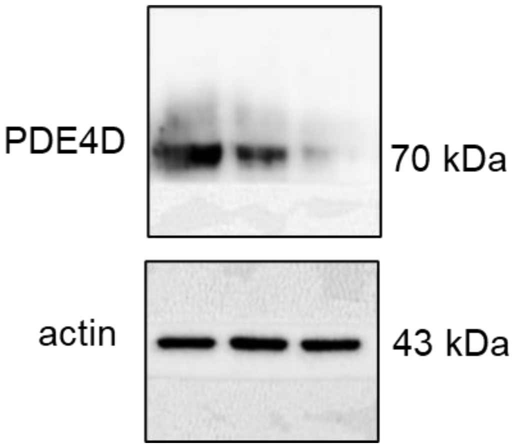

Fig. S9E

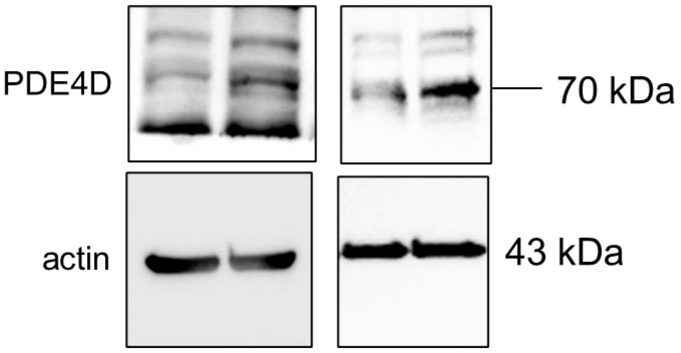

Supplement: Supplementary file 2 — Source Data for Western Blotting [file 41419_2024_6814_MOESM2_ESM.pdf]
